# From Plant to Chemistry: Sources of Active Opioid Antinociceptive Principles for Medicinal Chemistry and Drug Design

**DOI:** 10.3390/molecules28207089

**Published:** 2023-10-14

**Authors:** Rita Turnaturi, Silvia Piana, Salvatore Spoto, Giuliana Costanzo, Lorena Reina, Lorella Pasquinucci, Carmela Parenti

**Affiliations:** 1 Department of Drug and Health Sciences, Medicinal Chemistry Section, University of Catania, Viale A. Doria 6, 95125 Catania, Italy; silviapiana@outlook.com; 2 Department of Drug and Health Sciences, Section of Pharmacology and Toxicology, University of Catania, 95125 Catania, Italy; salvospoto12@icloud.com (S.S.); cparenti@unict.it (C.P.); 3 Department of Biomedical and Biotechnological Sciences, University of Catania, Via Santa Sofia 97, 95123 Catania, Italy; giuliana.costanzo93@gmail.com; 4 Postgraduate School of Clinical Pharmacology, Toxicology University of Catania, Via Santa Sofia n. 97, 95100 Catania, Italy; lorena.reina@hotmail.it

**Keywords:** opioid receptors, analgesic, pain, drug design, semisynthesis

## Abstract

Pain continues to be an enormous global health challenge, with millions of new untreated or inadequately treated patients reported annually. With respect to current clinical applications, opioids remain the mainstay for the treatment of pain, although they are often associated with serious side effects. To optimize their tolerability profiles, medicinal chemistry continues to study novel ligands and innovative approaches. Among them, natural products are known to be a rich source of lead compounds for drug discovery, and they hold potential for pain management. Traditional medicine has had a long history in clinical practice due to the fact that nature provides a rich source of active principles. For instance, opium had been used for pain management until the 19th century when its individual components, such as morphine, were purified and identified. In this review article, we conducted a literature survey aimed at identifying natural products interacting either directly with opioid receptors or indirectly through other mechanisms controlling opioid receptor signaling, whose structures could be interesting from a drug design perspective.

## 1. Introduction

Modification plant-derived active principles have been widely used in the treatment of diseases throughout human history [1]. Phytochemicals present in herbs continue to be actively investigated not only directly as therapeutic agents but also as prototype lead compounds for the development of new synthetic or semisynthetic drugs. In fact, when isolated from their source organisms, natural products could possess suboptimal pharmacological properties including poor pharmacokinetics, rapid metabolism, and low solubility or chemical stability [2]. Thus, the investigation into the chemical composition of medicinal plants and secondary metabolites, but especially (most of all) the structural modification of natural bioactive components, is a dynamic research field worldwide and plays an integral role in the process of drug discovery [3]. The literature is abundant with examples wherein the modification of a natural product has led to the development of a novel molecule drug with higher potency, a longer duration of action, and a better drug delivery method having minimal toxicity effects [4].

Taxol^®^ (*Taxus baccata*, Taxaceae) has been used to treat over one million patients, making it one of the most widely employed antitumoral drugs. It was the first agent described in the literature targeting the disruption of microtubule dynamics, thus inducing mitotic arrest and cell death [5]. Dimethyl fumarate (DMF) is one of the earliest discovered inducers of the KEAP1/NRF2 pathway, which regulates the expression of networks of genes that encode proteins with versatile cytoprotective functions, and it has essential roles in the maintenance of redox and protein homeostasis, mitochondrial biogenesis, and the resolution of inflammation [6]. The origins of the development of DMF as a drug date back to the use of the plant *Fumaria officinalis* (Papaveraceae) in traditional medicine [7,8]. More recently, a DMF formulation developed by Biogen has been tested in other immunological disorders, with successful phase III trials in multiple sclerosis [9], leading to its approval by the FDA and EMA in 2013. Thymol is a major component of essential oils from many plants. This natural phenol is not only clinically relevant as an anti-microbial, antioxidant, and anti-inflammatory agent, but it is also a privileged scaffold, which is diversified in natural sources. Evidence abounds linking the overall bioactivity of thymol to its monoterpene nucleus, specifically the hydroxyl (-OH) substituent on C1 carbon. This scaffold acts as an obligatory template for scheming and arriving at designing some newer drug-molecules with potential biological activities [10].

The search for active principles with analgesic properties has always been a requirement for man, and nature has proven to be an inexhaustible source. Opium has been used for pain management until the 19th century, when individual components, such as morphine, codeine and thebaine, were purified and identified. Morphine and its derivatives act through the mu opioid receptor (MOR), delta opioid receptor (DOR), and kappa opioid receptor (KOR) with differences in potency and selectivity [11]. An extensive drug discovery program based on morphine structural simplification or complication allowed for the development of compounds—some of which are currently used in clinic practice. Thus, several semisynthetic or synthetic compounds based on natural pharmacophores were introduced into the market. From these analgesic substances, the medicinal chemistry drug discovery approach was predominantly based on a rational drug design and completely novel synthetic efforts [12], with the aim of reducing struggles with side-effect profiles. For instance, to overcome MOR agonists’ adverse effects, a pioneering drug discovery approach focused on the synthesis of selective KOR and DOR agonists [11]. The dysphoric effect of KOR agonists and the weak effect of DOR agonists to counteract nociceptive pain led to an abrupt halt in their development, laying the foundations for the new medicinal chemistry paradigm “one molecule, multiple targets” [13,14]. The multitarget opioid approach, as a strategy to overcome typical side effects associated with selective opioid agonists, was supported by demonstrated physical and functional modulatory MOR/DOR and MOR/KOR interactions and their co-localization [15]. Recently, a new approach in the design of more tolerated drug candidates was based on the concept of biased agonists [16]. Moreover, apart from rational drug design and completely novel synthetic efforts, natural products are still being investigated for novel chemical structures [17] that could interact either directly with opioid receptors or indirectly through other mechanisms controlling opioid receptor signaling; this could be interesting from a drug design perspective.

## 2. Method Section

We have therefore focused on the search for natural analgesic compounds and their undertaken structural modifications to improve their profile. This review included the active principles or plant extracts that interact directly or indirectly with the opioid system. The search for appropriate papers for the purpose of this review was performed by using the internet sources MEDLINE-PubMed and EMBASE. The first step in the search was to collect all relevant articles by using different combinations of the following keywords: natural plants, opioid, pain, natural products, medicinal plants. The databases were searched for studies conducted in a period up to 2010. The papers were then screened to include any published paper that evaluated the use of natural products for pain in order to identify scaffolds with structural diversity and various bioactivities that could be directly developed or used as starting points for their optimization into novel drugs.

All electronic search titles, selected abstracts, and full-text articles were independently reviewed by four reviewers. After the removal of duplicates, citations were limited to animal studies, leading to the identification of about thirty natural sources. The second step of the literature search was performed using the following keyword combinations: principle active name, pain, semisynthetic, opioid, derivatives, and analogues. Meta-analyses, abstracts, conference proceedings, editorials/letters, and case reports were excluded. A systematic screening of the articles was performed according to the criteria of a) any biological activity that was the effect of natural products or their active principles on nociception based on animal models (acetic acid-induced abdominal writhing, formalin-induced nociception, orofacial formalin-induced nociception tests, chronic muscle pain tests, tail flick tests, hot plate tests, tail immersion tests, and von Frey tests) and their antinociceptive mechanisms of action; and b) plant material and chemical elucidation. In Table 1, the natural plant source, active principles, and synthetic analogues with tested antinociceptive activities are reported.

## 3. Sources of Active Opioid Antinociceptive Principles

### 3.1. Salvinorin A

**Figure 1:**
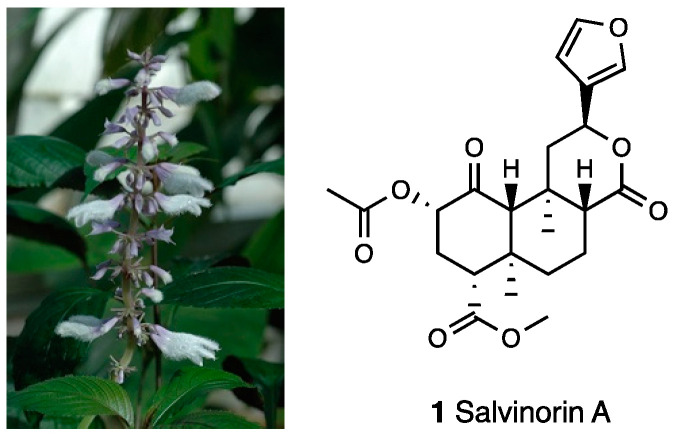
*Salvia divinorum* and its major active principle, Salvinorin A structure (**1**).

Salvinorin A (**1**, Figure 1), the major constituent of *Salvia divinorum* Epling and Jativa-M (Lamiaceae, Figure 1), is a neoclerodane diterpene notable for its lack of positive charge nitrogen, which is a crucial structural requirement for opioid receptor interaction [18,19].

**Figure 2:**
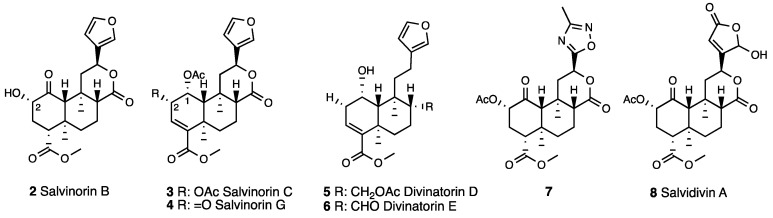
*Salvia divinorum* constituents’ structures: Salvinorin B, Salvinorin C, Salvinorin G, Divinatorin D, Divinatorin E, oxadiazole analogue, and Salvidivin A (**2**–**8**).

Salvinorin A binds selectively to KOR with K_i_ values of 4.3 and 16 nM (in brain and cloned receptors, respectively) and activates KOR with EC_50_ of 1.03 and 290 nM in the adenylyl cyclase and [^35^S]GTPγ assays [20,21,22]. Salvinorin A was efficacious against various pain animal models [23,24,25]. Intrathecally (i.t.) injected in mice, it reduced tail flick latencies with an ED_50_ of 20.93 nM. This antinociceptive effect was reversed by KOR antagonist nor-BNI, but it was not affected by the MOR or DOR antagonists (β-FNA and naltrindole) pre-treatment. Salvinorin A similarly reduced hot plate latencies and the number of writhes in acetic acid-induced abdominal constriction in mice. In these behavioral models, Salvinorin A was a short acting agent, given the fact that the effect disappeared 20 min after its administration. Other naturally occurring constituents were isolated from *Salvia divinorum*. Salvinorin B [26] (**2**, Figure 2), devoid of MOR, DOR, and KOR affinity, resulted from C-2 acetate removal. The C-1 acetylated derivative Salvinorin C (**3**, Figure 2) (K_i_^KOR^ = 1022 nM) and its C-2 deacetylated analogue Salvinorin G (**4**, Figure 2) reported a decreased KOR affinity, which was ultimately abolished after C-1 acetate hydrolysis [27,28,29]. The C-ring lactone removal in Divinatorins D and E (**5** and **6**, Figure 2) (K_i_^KOR^ = 230 nM and 418 nM, respectively) led to a reduction in KOR affinity in comparison to Salvinorin A [30], whereas oxadiazole **7** (Figure 2, K_e_^KOR^ = 360 nM) and Salvidivin A (Figure 2, K_e_^KOR^ = 440 nM), C-12 furan-modified derivatives, were identified as the first naturally occurring KOR antagonists [31].

**Figure 3:**
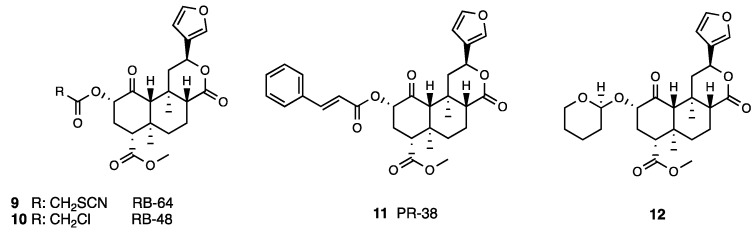
Structures of C-2 modified Salvinorin A analogues (**9**–**12**).

Several Salvinorin A derivatives were synthesized by modifying the acetoxy substituent at C-2 to establish the steric and physicochemical properties of this site for KOR interaction and, above all, minimize the Salvinorin A metabolism into the inactive Salvinorin B [32,33]. An improved KOR efficacy profile was reported in derivative MOM-Sal B obtained through C-2 methoxymethyl introduction into the Salvinorin B template [34]. MOM-Sal B in competition binding experiments performed in CHO cells expressing human KOR showed a three times higher KOR affinity (0.4 vs. 1.3 nM) and activated KOR with a seven times increased potency (EC_50_ = 0.6 nM E_MAX_ = 98%) than Salvinorin A (EC_50_ = 1.3 nM E_max_ = 106%) as detected by [^35^S]GTPγ binding experiments. The C-2 structural modification in MOM-Sal B produced a long lasting (120 min) effect, which was nor-BNI reversed in a rat hot plate test [35]. Mesyl Sal B [36], obtained via isosteric replacement of the C-2 acetate group with a sulfonate ester, retained the KOR efficacy profile of the lead Salvinorin A. The 22-thiocyanatosalvinorin A RB-64 and the 22-chlorosalvinorin A RB-48 (**9** and **10**, Figure 3) were investigated in vitro and in vivo [37].

Both compounds showed equivalent affinities (K_i_^KOR^ = 0.59 and 2.1 nM for RB-64 and RB-48, respectively) and were similar to that of Salvinorin A. RB-64 and RB-48 were highly potent KOR agonists with EC_50_ values of a hundred- and eighty-times higher (EC_50_ = 0.077 and 0.19 nM; E_max_ = 95 and 85% for RB-64 and RB-48, respectively). Moreover, White et al. demonstrated that both derivatives are potent in activating G-protein signaling (EC_50_ = 5.29 and 8.82 nM for RB-64 and RB-48, respectively) and possess a high bias degree (EC_50_ = 391 and 143 nM for RB-64 and RB-48, respectively) [38].

A series of Salvinorin A derivatives modified at C-2 with reactive Michael acceptor groups has been also synthesized [39]. Synthesized acryloyl and cinnamic acid derivatives showed a significant KOR affinity with a K_i_ range from 6 to 581 nM, but some of them showed MOR and KOR affinity. In particular, the acid cinnamic derivative PR-38 (**11**, Figure 3) [40], with K_i_ values at MOR and KOR of 52 and 9.6 nM coupled to a potent dual MOR/KOR agonist profile, resulted a potential anti-inflammatory and analgesic agent for gastrointestinal inflammation [41] in a mouse model of hypermotility, diarrhea, and abdominal pain. Moreover, in the behavioral model based on the i.c. mustard oil instillation, i.p., i.c., and p.o. PR-38 administration in mice attenuated colitis, showing a significant anti-scratch activity reversed by β-FNA [42].

**Figure 4:**
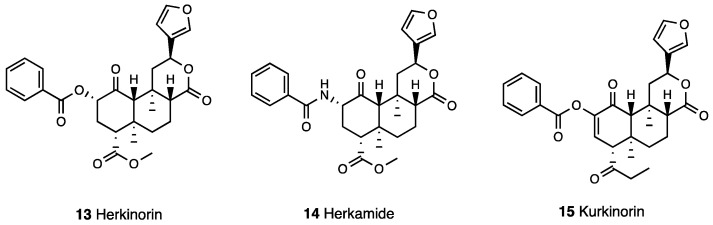
Structures of Herkinorin (**13**), Herkamide (**14)** and Kurkinorin (**15**).

Prisinzano et al. [43] synthesized a β-tetrahydropyranyl ether of Salvinorin B (**12**, Figure 3) that showed a slightly higher affinity (K_i_^KOR^ = 6.2 nM) than Salvinorin A and had a similar potency (EC_50_ = 60 nM vs. 40 nM). The tetrahydropyran group at C-2 conferred to the compound potent antinociceptive effect in an acute thermal pain assay (5 times higher than the KOR agonist U50,488 and equipotent to that of Salvinorin A), and it was also able to reduce both phase 1 and 2 of formalin-induced inflammatory pain and paclitaxel-induced neuropathic pain. However, the compound showed the classical conditioned place aversion of KOR agonists. A 47-time loss in KOR affinity (K_i_^KOR^ = 90 nM) and a 27-time increase in MOR affinity (K_i_^MOR^ = 12 nM) was reported for Herkinorin (**13**, Figure 4) in comparison to Salvinorin A, obtained via replacement of the acetoxy with a phenoxy group at C-2 [44].

Herkinorin, a potent MOR/KOR agonist, is the first example of a non-nitrogenous MOR agonist that does not promote β-arrestin 2 recruitment and MOR internalization [45]. What has also been evaluated is the effect of electron-donating or -withdrawing groups at the benzyl ring or its replacement with heteroaromatic esters, as well as the spacer introduction between the carbonyl and the phenyl ring of Herkinorin [46,47]. Through these studies, a *N*-benzamido derivative of Herkinorin, named Herkamide (**14**, Figure 4), emerged with improved MOR affinity and selectivity (K_i_ = 3.1 nM) and increased MOR agonist potency.

The C2–C3 unsaturation introduction in the Herkinorin structure led to Kurkinorin [48] (**15**, Figure 4). This conformational restriction affects the orientation of the C-2 substituent for the interaction of MOR and KOR, conferring a decreased KOR activity (EC_50_ > 10,000 nM) to Kurkinorin but improved MOR (EC_50_ = 1.2 nM) and DOR (EC_50_ = 74 nM) activity. In a hot water tail flick test, Kurkinorin elicited an antinociceptive effect, which was naloxone reversed and, in a regimen of repeated administration, maintained its effect until the ninth day of administration, resulting in a low-tolerance-inducing liability. The interesting analgesic profile of Kurkinorin, coupled with a reduced incidence of sedation in comparison to morphine—as assessed by a rotarod behavioral assay—was related to the low propensity of the compound to promote β-arrestin 2 recruitment, resulting in a biased MOR agonist. In Table 2, the most important information on Salvinorin A and its derivatives is reported.

Other synthetic Salvinorin A derivatives were also obtained from structural modifications of the tricyclic *trans*-decalin core. Modification of the ketone in the C-1 position of Salvinorin A clarified its involvement in KOR interaction [28]. For instance, the reduction of C-1 ketone to an α-alcohol (**1a**) or its complete removal (**1b**) caused both a diminution in KOR affinity and shifted the KOR profile from full agonism to antagonism [49]. Analogously, the introduction of a 1,10-alkene (**1c**) lowered efficacy across all opioid receptors and a further oxidation of the C-2 position (**1d**) produced an α,β-unsaturated derivative with a KOR antagonist profile. These findings, suggesting a dissimilar binding mode of C-1 deoxo and 1,10-dehydro analogues to their C-1 keto congeners, were also reported for Herkinorin, whose C-1 ketone modifications shifted the functional profile from MOR agonism to antagonism.

**Figure 5:**
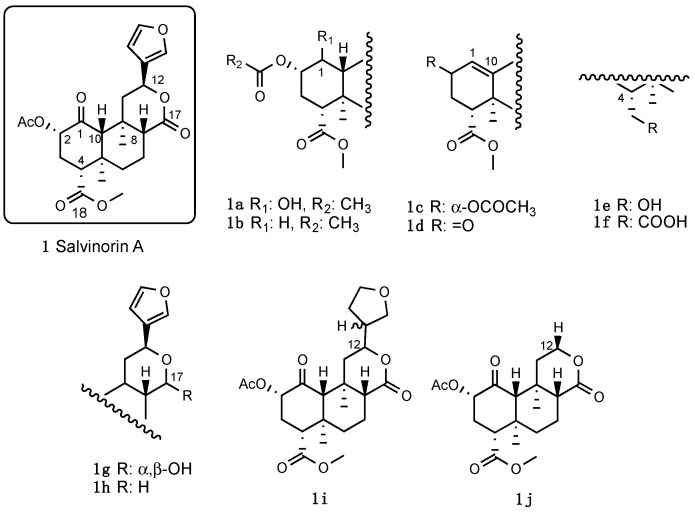
Salvinorin A derivatives obtained through structural modifications of the tricyclic *trans*-decalin core (**1a**–**1d**), 4-carbomethoxy group (**1e**, **1f**), C-17 carbonyl group (**1g**, **1h**) and C-12 furan ring (**1i**, **1j**).

The structural modifications of the 4-carbomethoxy group [50,51], such as its reduction (**1e**) or hydrolysis (**1f**), conducted to derivatives with lower efficacy at KOR, reflecting the importance of the C-18 hydrogen bond acceptor for interactions with the KOR hydrophobic binding pocket. Modifications of the C-17 carbonyl in C-ring through its reduction (**1g**) or complete removal (**1h**) were also unfavorable. Additionally, modifications of the C-12 furan ring through its reduction (**1i**), removal (**1j**), or replacement with other heterocycles were deleterious [52,53,54,55] (Figure 5).

**Figure 6:**
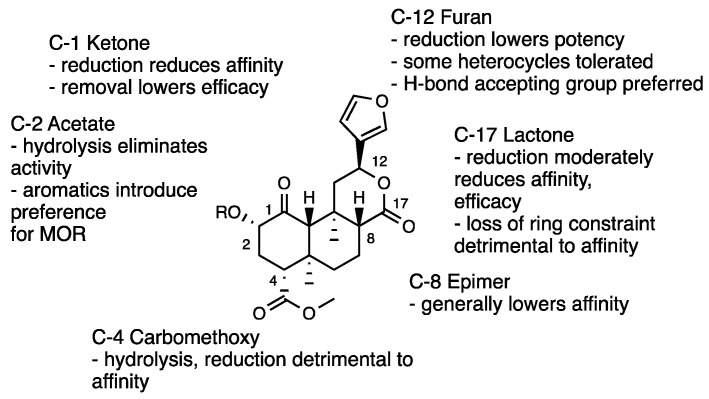
Salvinorin A SAR for the KOR activity.

In Figure 6, the most relevant Salvinorin A SAR for the KOR activity are summarized.

### 3.2. Mitragynine

*Mitragyna speciosa* Korthals is a tree of the Rubiaceae family, which is indigenous to Southeast Asia. Known as “Kratom” in Thailand and “Biak-Biak” in Malaysia, the leaves have been traditionally used by natives for their opium-like effect and coca-like stimulant ability to combat fatigue and enhance tolerance to hard work [56,57].

**Figure 7:**
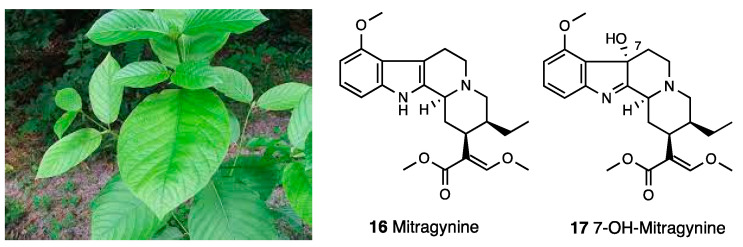
*Mitragyna speciosa* and its major active principles Mitragynine (**16**) and 7-OH-mitragynine (**17**).

Mitragynine and its naturally occurring oxidation product, 7-hydroxy-mitragynine (7-OH-mitragynine), were isolated from *Mitragyna speciosa* (**16** and **17**, Figure 7). They are monoterpene indole alkaloids, structurally not closely related to morphine. In the homogenates of a guinea pig brain, an interesting affinity profile towards MOR, DOR, and KOR was established for mitragynine (K_i_^MOR^ = 7.2 nM, K_i_^DOR^ = 60 nM, K_i_^KOR^ > 1000 nM) and 7-OH-mitragynine (K_i_^MOR^ = 13 nM, K_i_^DOR^ = 155 nM, K_i_^KOR^ = 123 nM) [58]. In electrically stimulated GPI preparations, mitragynine and 7-OH-mitragynine were full agonists with 1/4 potency and 10-fold greater potency than morphine, respectively [59]. Mitragynine and 7-OH-mitragynine displayed centrally mediated (supraspinal and spinal) antinociceptive activity in various pain models [60,61]. Through hot plate and tail pinch tests, a dose-dependent antinociceptive activity—completely abolished by both s.c. and i.c.v naloxone—was recorded for mitragynine, which was administered in a range of 5.0–30 mg/kg, i.p. and 1.0–10 pg/mouse, i.c.v. [62,63]. Different opioid receptor subtypes’ involvement in the antinociceptive effect of mitragynine was subsequently confirmed. I.c.v. mitragynine-induced antinociception was antagonized by i.c.v. naloxonazine, a selective MOR antagonist, and by the co-administration of i.c.v. naltrindole, a DOR-antagonist, in tail pinch and hot plate tests. Nor-BNI antagonized i.c.v. mitragynine-induced antinociception only in the tail pinch test [64]. A recent study shows that mitragynine and the oxidized analog 7-OH-mitragynine are partial agonists of MOR (EC_50_ = 339 nM, E_max_ = 34% and EC_50_ = 34.5 nM, E_max_ = 47%, respectively) and competitive antagonists of KOR and DOR. It was also shown that mitragynine and 7-OH-mitragynine are G-protein-biased agonists of MOR, which do not recruit β-arrestin, following receptor activation.

Descending noradrenergic and serotonergic system involvement in the antinociceptive activity of mitragynine in mechanical noxious stimulation tests was also demonstrated, since a slight and significant increase in the expression of the immediate early gene c-Fos was observed following acute and chronic treatment in male Wistar rats [65].

Different evidence suggested mitragynine was useful for inflammatory conditions, since it inhibited COX-2 mRNA and protein expression, as well as PGE2 formation, in a dose-dependent manner in LPS-stimulated RAW264.7 macrophage cells. Mitragynine also affects COX-1 protein expression [66]. Additionally, i.p. administration of the methanolic extract of *Mitragyna speciosa* has anti-inflammatory properties that are able to inhibit the development of a carrageenan-induced paw edema [67].

**Figure 8:**
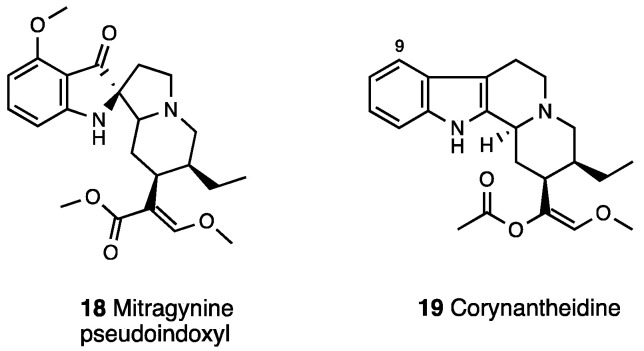
Other constituents of *Mitragyna speciosa* preparations (**18** and **19**).

Natural mitragynine products are represented by diverse indole cores (indole, indolenine, and spiro pseudoindoxyl) with opioid activity [68]. Other than the oxidize analogue 7-OH-mitragynine (indolenine core), other constituents of *Mitragyna speciosa* preparations are mitragynine pseudoindoxyl (**18**, Figure 8), a rearrangement product of 7-OH-mitragynine with a spiro-pseudoindoxyl core, and corynantheidine (**19**, Figure 8), a 9-demethoximitragynine that, as assessed by GPI, is a MOR antagonist [69].

Mitragynine pseudoindoxyl produced a 10-fold higher analgesic effect than mitragynine, with an MOR and DOR affinity profile similar to DAMGO (pIC_50_ = 8.18 vs. 8.77) and DPDPE (pIC_50_ = 9.55 vs. 8.90), respectively, but a negligible affinity at KOR (pIC_50_ = 6.88) and an efficacy profile of a MOR agonist/DOR antagonist as assessed by GPI (pD_2_ = 8.96), MVD (pD_2_ = 7.40), and [^35^S]GTPγ functional assays [70]. Mitragynine pseudoindoxyl represents an example of a mixed MOR agonist/DOR antagonist ligand that failed to recruit β-arrestin 2. In fact, it developed analgesic tolerance more slowly than morphine and showed effects of limited physical dependence, respiratory depression, constipation, and rewarding or aversive behavior in a conditioned place preference paradigm. In Table 3, the most important information on mitragynine and its derivatives is reported.

**Figure 9:**
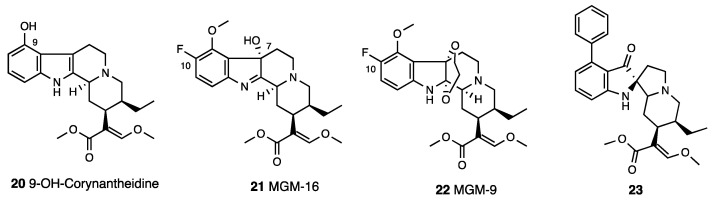
Structures of 9-hydroxycorynantheidine (**20**), MGM-16 and MGM- (**21** and **22**), and 9-phenyl analogue (**23**).

Mitragynine and 7-OH-mitragynine structural modifications identified the major sites for opioid receptor interaction [71,72]. The methoxy group in C-9 is crucial for antinociception. Indeed, the demethoxymitragynine derivative, corynantheidine, lost opioid agonistic activity. The 9-desmethyl derivative, known as 9-hydroxycorynantheidine (**20**, Figure 9), showed significant affinity for MOR (pK_i_ = 7.92) and inhibited the electrically induced twitch contraction in GPI, indicating its partial agonist property (pD_2_ = 6.78) [59,73]. The elongation of the alkoxy chain in C-9 with superior homologues led to compounds able to inhibit electrically induced contraction in GPI, an effect naloxone insensitive. Thus, the C-9 position on the corynantheidine scaffold is important for the intrinsic activity of these compounds.

Building upon the 46-fold potency of 7-OH-mitragynine, the importance of C-7 for opioid receptor interactions was underlined. MGM-16 (**21**, Figure 9) was suitable for acute and chronic pain management [74]. In a competition binding assay, MGM-16 showed high MOR, DOR, and significant KOR affinity, and in [^35^S]GTPγS binding, GPI and MVD assays were potent MOR/DOR agonists. This efficacy profile was also confirmed in vivo with a mouse tail flick test. MGM-16, p.o. and s.c. administered, produced a dose-dependent antinociception, significantly and partially reversed by β-FNA and naltrindole, respectively. Moreover, in SNL mice, MGM-16 produced a significant antiallodynic effect reversed by selective MOR and DOR antagonists. MGM-9 (**22**, Figure 9), a C-7 ethylene glycol-bridged and C-10-fluorinated derivative of mitragynine, showed a MOR/KOR agonist efficacy profile (K_i_^MOR^ = 7.30 nM, K_i_^KOR^ = 18 nM) [75].

Structural derivatization of the *N*_b_ lone pair and β-methoxyacrylate moiety were conducted to mitragynine derivatives with no or weak opioid receptor activity [72], suggesting β-methoxyacrylate residue was needed for opioid receptor interactions and confirming the *N*_b_ lone pair essential feature of the opioid pharmacophore [76].

Váradi et al. [77] designed pseudoindoxyl and semisynthetic analogues. Differently from other derivatives in which C-9 mitragynine modifications altered efficacy at MOR, C-9 mitragynine pseudoindoxyl substituents maintained a full MOR functional profile. C-9-substituted derivatives were DOR antagonists, except for 9-phenyl analogue (**23**, Figure 9), which was a DOR agonist. The 9-phenyl analogue is a MOR agonist/DOR antagonist, which produces a similar antinociception of the parent mitragynine pseudoindoxyl with a low incidence of side effects.

### 3.3. Collybolide

**Figure 10:**
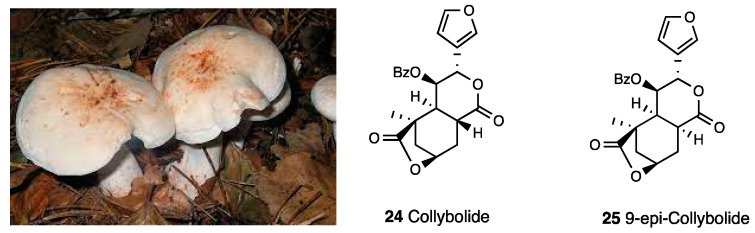
*Collybia maculate* and its most relevant active principles Collybolide (**24**) and 9-epicollybolide (**25**).

The natural product collybolide (**24**, Figure 10), extracted from the fungus *Collybia maculate* (Figure 10), is a sesquiterpene sharing a furyl-δ-lactone core with Salvinorin A.

Other isolated constituents include 9-epicollybolide (**25**, Figure 10), isocollybolide, and neocollybolide, as well as a few other collybolide-like sesquiterpenes. Collybolide is a highly potent and selective KOR agonist. In competition binding experiments, performed in HEK-293 cells expressing human MOR, DOR, or KOR, collybolide binds KOR (K_i_ = 10^−10^ M) with a high degree of selectivity. Collybolide dose-dependently increases [^35^S]GTPγ binding (EC_50_ of 2 nM, E_max_ of 124%) and decreases adenylyl cyclase activity with an IC_50_ of 0.9 nM. Moreover, as tested by an ERK1,2 phosphorylation assay, collybolide was a biased KOR agonist. Collybolide decreased tail flick latencies in mice and showed the same effects in a forced swim test and an elevated plus-maze test of Salvinorin A. Collybolide differs from Salvinorin A in blocking chloroquine-mediated itch. Analogous investigations performed with the C9 collybolide epimer showed the importance of this position for KOR interaction, since 9-epicollybolide featured reduced KOR binding, efficacy, and signaling [78,79].

### 3.4. Corydine, Corydaline, Dehydrocorybulbine (DHCB) 

**Figure 11:**
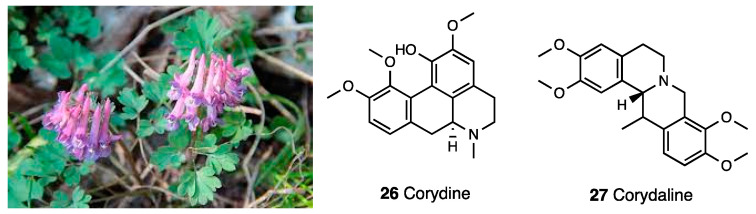
*Corydalis yanhusuo.* W.T. Wang and its most relevant active principles Corydine (**26**) and Corydaline (**27**).

*Corydalis yanhusuo* W.T. Wang (Figure 11) is a perennial herb in the Papaveraceae family. It was investigated in different animal models of acute, inflammatory, and chronic pain.

Kaserer et al. [80], using a collection of models for pharmacophore-based virtual screening, identified structural analogues reported as natural products isolated from *Corydalis* and *Berberis* species [81,82,83], such as corydine and corydaline (**26** and **27**, Figure 11). The in vitro screening established for both compounded a functional profile of biased MOR agonists. Through competition binding experiments performed in CHO cells expressing human MOR, corydine and corydaline were able to displace more than 50% of [^3^H]-DAMGO binding showing a moderate but selective MOR affinity (K_i_ = 2.82 and 1.23 μM, respectively). By using the same cell line, both compounds increased the [^35^S]GTPγ binding in a concentration-dependent manner, becoming full MOR agonists with corydine (EC_50_ = 0.51 μM) that was 3 times more potent than corydaline (EC_50_ = 1.50 μM); however, both were 34 times less potent than DAMGO (MOR reference compound). Unlike DAMGO, both compounds failed to promote β-arrestin 2 recruitment as assessed in the PathHunter β-arrestin 2 recruitment assay. In Table 4, the most important information on *Corydalis yanhusuo* active principles is presented.

AN interesting functional profile delineated in vitro for corydine and corydaline was also evaluated in vivo through the writhing test. Compared to morphine, corydine and corydaline administered in mice at doses of 5 and 10 mg/kg s.c. were 10 and 20 times, respectively, less effective in reducing the number of writhes. Their antinociceptive effect was naloxone reversed and, at tested doses, corydine and corydaline did not show alterations for locomotor activity and sedation in mice.

**Figure 12:**
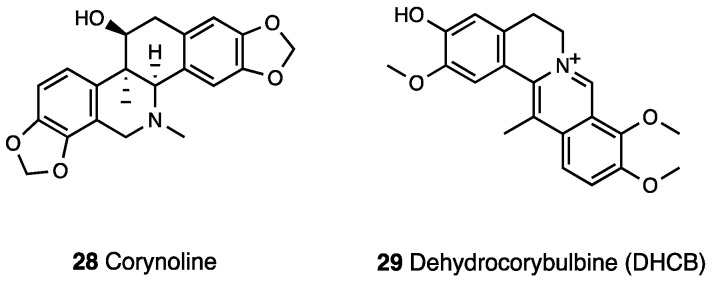
Corynoline (**28**) and dehydrocorybulbine (**29**) structures.

The antinociceptive effect of corynoline (**28**, Figure 12), a phytochemical compound isolated from *Corydalis bungeana* Turcz., has been evaluated in several pain models [84]. Corynoline was found to effectively suppress nociception in hot plate, tail immersion, glutamate, acetic acid, and capsaicin tests in mice. Corynoline was also observed to inhibit carrageenan-induced inflammation.

The water extract of the tuber of *Corydalis yanhusuo* W.T. Wang contains several alkaloids [85,86] such as berberine [87], palmatine, columbamine, and glaucine [88]. Injected in mice in doses of a 100–500 mg/kg range, it significantly increased tail flick latencies at the highest tested dose without affecting locomotor activity, as assessed with a rotarod test [89]; a formalin test at a dose of 200 mg/kg reduced both phase I and II. Additionally, an extract injection produced a significant antiallodynic and antihyperalgesic effect in the SNL model of neuropathic pain at the same dose. Moreover, the extract could present advantages over morphine in chronic pain treatment since, after repeated administration, it did not lead to tolerance development. Thus, the antinociceptive properties of the water extract of *Corydalis yanhusuo* W.T. Wang could be the result of additive or synergistic effects of all alkaloids and other unidentified active components contained in it.

By screening on cells expressing MOR, Zhang et al. [90] isolated a fraction of *Corydalis yanhusuo* that induced a significant receptor-dependent calcium mobilization. After efficient HPLC purification, the active component—dehydrocorybulbine (DHCB) (**29**, Figure 12)—was isolated, and its structure was established by UV, mass spectroscopy, NMR, and X-ray crystallography. DHCB behaves as a weak MOR agonist and dopamine receptor antagonist. In a tail flick test, the antinociceptive effect of DHCB at the highest dose (40 mg/kg, i.p) was similar to that of morphine, and indeed DHCB produced a naloxone-resistant antinociceptive response consistent with its weak MOR affinity.Indeed, its dopamine receptor affinity (more than one hundred times higher than the MOR affinity) and its involvement in the DHCB effect was established in the same experimental model, via pretreatment with the D2 receptor agonist quinpirole, which affected the tail flick response. Analogous results resulted from performing the tail flick test in D2 receptor KO mice. Consistent with its mechanism of action, DHCB did not show tolerance-inducing capabilities in a regimen of repeated administration (10 mg/kg daily for 7 consecutive days). DHCB was also effective in both phase I and II of formalin- induced inflammatory pain and injury-induced neuropathic pain.

**Figure 13:**
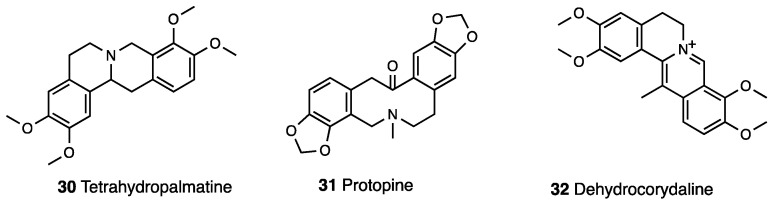
Structures of primary active components (**30**–**32**) of *Corydalis yanhusuo*.

In another study [91], the primary active components of *Corydalis yanhusuo*, L-tetrahydropalmatine (l-THP), protopine, dehydrocorydaline (**30**–**32**, Figure 13), and corydaline (**27**) were tested. Through whole-cell voltage-clamp experiments, where all four alkaloids exhibit strong inhibitory effects upon Nav1.7. and showed antinociceptive effects in both phase I and phase II, with l-THP more efficacious in phase I and protopine more efficacious in phase II of the formalin test.

In earlier studies, a relatively high D1 and D2 receptor and low 5-HT_1A_ binding affinity [92] and a dopamine D2 receptor-mediated antinociception via rat radiant tail flick test was established for l-THP [93]. l-THP was also investigated in a mouse model of oxaliplatin-induced neuropathic pain [94]. The alkaloid produces a robust dose-dependent (1–4 mg/kg) antihyperalgesic effect primarily mediated by D_1_ receptors since the selective D_1_ receptor antagonist SCH23390 significantly attenuated the antihyperalgesic effects of l-THP. Analogous evidence emerged in complete Freund’s adjuvant-induced inflammatory pain [95]. Importantly, in the regimen of repeated daily l-THP treatment with 4 mg/kg, a dose that completely reversed mechanical hyperalgesia, the compound maintained its antihyperalgesic effect without significant antinociceptive tolerance development over a period of 10 days. The antihyperalgesic effects of pre- and post-treatment with l-THP in the bee venom pain model has also been demonstrated [96]. Through immunohistochemistry studies, l-THP-induced antihyperalgesic effects have been also related to its capability to down-regulate P2X3 and TRPV1 protein receptor expression in the spinal cord, whose levels were markedly increased following an s.c. bee venom injection. Moreover, l-THP did not significantly alter the locomotor activity in mice. Kang et al. [97] designed a study that was performed to elucidate the l-THP mechanism in the spinal cord and was related to its antinociceptive effects in acute and chronic pain [98]. Through formalin test, it was demonstrated the capability of i.p.-injected l-THP to counteract phase II of the formalin test, which, differently from phase I, is dependent on the combination of an inflammatory reaction in the peripheral tissue and functional changes in the dorsal horn of the spinal cord. To demonstrate the l-THP mechanism in the spinal cord, an in vivo model of mechanical allodynia, induced by spinal sigma1 receptor (Sig-1R) activation through i.t. administration once a day for 10 consecutive days of the Sig-1R agonist PRE084, was used. I.p. l-THP administration led to mechanical allodynia reduction that was Sig-1R activation induced. In CCI-rats, i.t. treatment with l-THP combined with the Sig-1R antagonist, BD1047, synergistically blocked mechanical allodynia, suggesting that l-THP modulates spinal Sig-1R activation.

**Figure 14:**
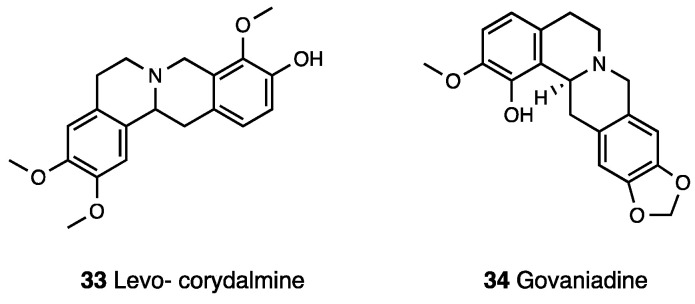
Levo-corydalmine (**33**) and Govaniadine (**34**) structures.

Obtained by replacing the methoxy group at the C-10 position of l-THP with a phenolic hydroxyl group, levo-corydalmine (**33**, Figure 14), antinociceptive effects and underlying mechanisms were evaluated in a model of neuropathic pain induced by vincristine [99].

In the same study, the levo-corydalmine capability to reduce the prevalence of atypical mitochondria induced by vincristine in both A- and C-fibers was demonstrated. Moreover, levo-corydalmine protects against nerve damage and attenuates vincristine-induced neuroinflammation by upregulating Nrf2/HO-1/CO to inhibit Cx43 expression. Several alkaloids from the *Corydalis govaniana* Wall with antioxidant, anticancer, and pain relief were isolated and characterized. Among them, the potential antinociceptive effect of govaniadine (**34**, Figure 14) was in vivo tested [100]. Similarly to diclofenac, govaniadine reduced nociception in a dose-dependent manner via acetic acid-writhing test, a pain model that implied the release of arachidonic acid and cyclooxygenase products. As presented by a docking study, govaniadine fit into the binding pocket of a COX-2 enzyme. I.p.-injected govaniadine elicited significant antinociception, which was naloxone-reversed in a hot plate test, suggesting a central effect for the alkaloid.

### 3.5. Essential Oil of Himenaea cangaceira 

**Figure 15:**
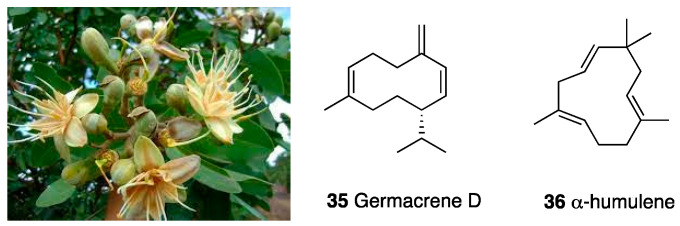
*Himenaea cangaceira* and structures of primary analgesic compounds (**35** and **36**).

Essential oil extracted from fresh leaves of *Himenaea cangaceira* (Figure 15) was investigated for its chemical composition and antinociceptive activity [101].

*Himenaea cangaceira*, distributed in Central and South America and in Brazil, is consumed for food and disease treatment [102]. In the essential oil, obtained via hydrodistillation and analyzed by GC-MS, 15 compounds, reported in the literature as biologically active, were found to have a prominent percentage (about 80%) of sesquiterpenes, such as (E)-caryophyllene, germacrene D, α-guaiene, β-elemene, α-copaene, and α-humulene. Germacrene D (**35**) is reported to be antitumoral and analgesic; α-guaiene is reported as an inhibitor of cyclo- and lipo-oxygenase and acetylcholinesterase; β-elemene and α-copaene are described for their antimicrobial activity; α-humulene (**36**) is detected for its anti-inflammatory, analgesic, and anti-allergic activity. Based on this evidence, the essential oil was in vivo tested for its potential analgesic effect. The essential oil was able to reduce the number of writhes in an acetic acid-induced pain model. Moreover, in both phase I and II of the formalin test, it reversed behavioral signs of inflammatory pain, suggesting the involvement of central and peripheral pathways and, given that naloxone reversed its effects in phase II, opioid receptor involvement.

### 3.6. Polymethoxyflavones of Ageratum conyzoides 

**Figure 16:**
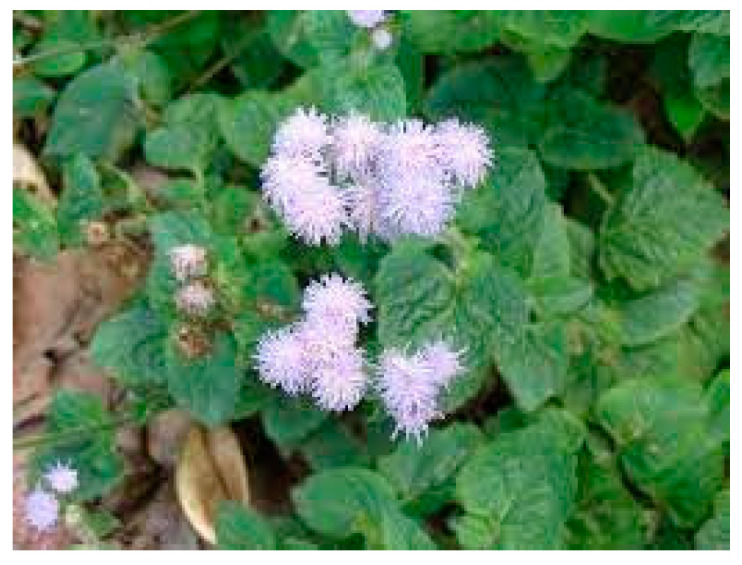
*Ageratum conyzoides*.

The annual herbaceous *Ageratum conyzoides* (Figure 16), widely distributed in tropical and subtropical regions, is used in popular Brazilian medicine to treat pain, fever, and inflammatory chronic disease such as rheumatoid arthritis.

**Figure 17:**
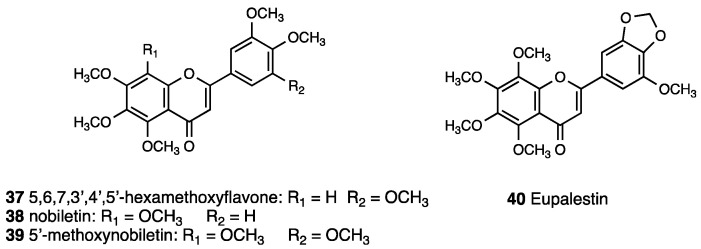
Major polymethoxyflavones (**37**–**40**) in *Ageratum conyzoides* standardized extract.

Literature data [103] reported significant effects of the water extract of *Ageratum conyzoides* leaves in a rat carrageenan assay [104], as well as reduction of acute and chronic pain effects of the ethanolic extract in cotton-induced granuloma and in formaldehyde-induced arthritis in rats. Chromenes, chromones, benzofurans, coumarins, monoterpenes, pyrrolizidine alkaloids, steroids, and flavonoids are the major phytochemicals found in *Ageratum conyzoides*. For all of them, a contribution to inflammation pathways was reported, with the exception of pyrrolizidine alkaloids, for which serious hepatotoxicity, carcinogenicity, genotoxicity, and teratogenicity was reported. A qualitative analysis of the standardized extract of polymethoxyflavones, performed through UHPLC/MS, detected 5,6,7,3′,4′,5′-hexamethoxyflavone, nobiletin, 5′methoxynobiletin, and eupalestin (**37**–**40**, Figure 17) as major peaks, and the absence of pyrrolizidine alkaloids was also detected [105].

I.p. administration of this standardized extract (10–300 mg/kg) reduced both neurogenic and inflammatory phases of formalin-induced nociceptive behavior, although its effect was marked in phase II. Moreover, at the highest tested dose, the standardized extract reduced paw edema induced by a formalin i.p. injection. In agreement with literature data [106], the extract reduced nociception elicited by the i.p. injection of PGE2. The essential oil of *Ageratum conyzoides*, obtained through steam distillation and characterized through GC-MS, contains 60 compounds [107]. In a CCI rat model of neuropathic pain, the essential oil (100 mg/kg) produced significant anti-hyperalgesic and anti-allodynic effects, which were naloxone reversed.

### 3.7. Brachydin A, Brachydin B, and Brachydin C

**Figure 18:**
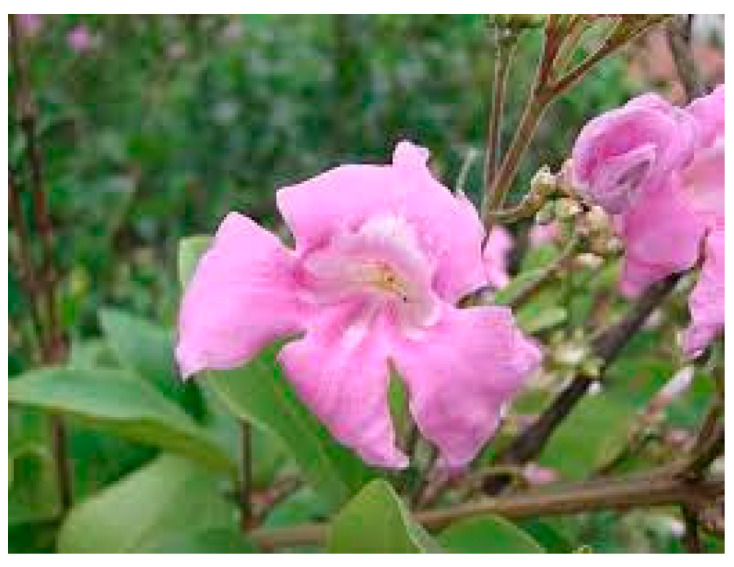
*Arrabidaea brachypoda* (D.C.).

*Arrabidaea brachypoda* (D.C.) (Figure 18) is a shrub native to the Brazilian region of Cerrado. It belongs to the Bignoniaceae family, which includes 120 genera and nearly 800 species of different plants scattered in tropical and subtropical regions worldwide. It is used in traditional medicine to treat pain and inflammation, and its roots are used for the treatment of joint pain in particular [108,109,110].

**Figure 19:**
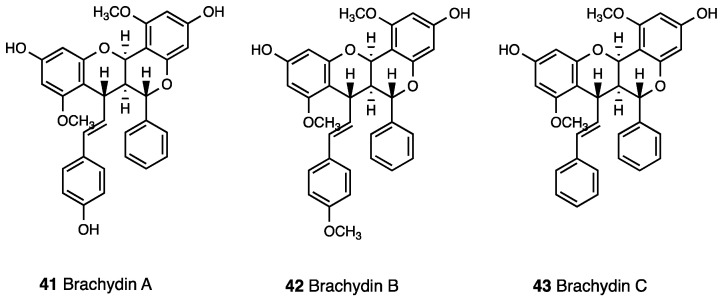
Major constituents (**41**–**43**) detected in the dichloromethane fraction extracted from the ethanolic root extract of *Arrabidaea brachypoda* (D.C.).

Extracts from the roots, leaves, and flowers [111] have been explored. The ethanolic extract of root, whose phytochemical analysis revealed the presence of flavonoids, saponins, coumarins, tannins, cardiac glycosides, steroids, and phenolic compounds, possess analgesic and anti-inflammatory activity which supports its traditional use [112]. In acetic acid-induced writhing test, the ethanolic extract, p.o. administered in mice in a dose range of 30–300 mg/kg, reduced the amount of writhing, and in a formalin test it was able to counteract phase II of inflammatory pain. Moreover, it significantly reduced carrageenan-induced paw edema and, in the rat model of peritonitis induced by LPS, it inhibited leukocyte recruitment into the peritoneal cavity in rats. In a later study, the dichloromethane fraction extracted from the ethanolic root extract was characterized and in vivo evaluated [113]. HPLC–PDA analysis [114] revealed only three major compounds that were identified and fully characterized as three unusual dimeric flavonoids: brachydin A, brachydin B, and brachydin C (**41**–**43**, Figure 19).

The antinociceptive effect was evaluated in formalin and hot plate tests (10–100 mg/kg p.o.) in mice, after verifying that the dichloromethane extract did not impair locomotor activity, through a rotarod apparatus. Differently from ethanolic extract, the dichloromethane extract induced antinociception during both neurogenic and inflammatory phases of the formalin test. Further investigation via hot plate test showed no antinociceptive effect of the extract vs. supraspinal response. It was also demonstrated that the dichloromethane extract was effective in reverse nociceptive behavior induced by methanol, an activator of TRPM8, and by acidified saline, which is an activator of ASIC, but it was ineffective in reverse nociceptive behavior induced by capsaicin and cinnamaldehyde (which are activators of TRPV1 and TRPA1, respectively). Moreover, complete naloxone-induced antinociceptive reversion revealed opioid receptor involvement in the antinociceptive effect of the dichloromethane extract. In vitro anti-inflammatory activity in the arthritic synoviocytes of the ethanolic extract, dichloromethane extract, and three dimeric flavonoids was evaluated [115]. In this investigation, ethanolic extract’s lack of effectiveness was probably because the test setup did not allow for the releasing of aglycones deriving from glucoronated forms of not only brachydins A, B, and C, but also other molecules of the extract. Dichloromethane extract instead showed an effect against the release of pro-inflammatory cytokine IL-6 as well as brachydins. However, brachydin A was less active than B and C alone, probably due to differences in polarity that can affect bioavailability and bioactivity.

### 3.8. Nuciferine and N-Nornuciferine

**Figure 20:**
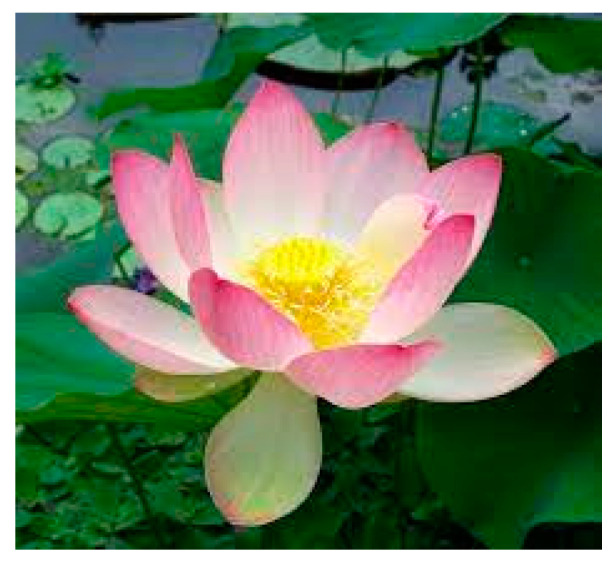
*Nelumbo nucifera* Gaertn.

*Nelumbo nucifera* Gaertn (Nymphaeaceae, Figure 20), known as sacred lotus, is an aquatic plant with a wide array of traditional, medicinal, and therapeutic uses [116,117] such as nervous disorder, high fever with restlessness, insomnia, hypertension, cancer, weakness, body heat imbalance [118], stress, depression, pain, and cognitive disorders [119,120,121]. Moreover, smoking the plant creates a feeling of well-being and controlled stress [122].

**Figure 21:**
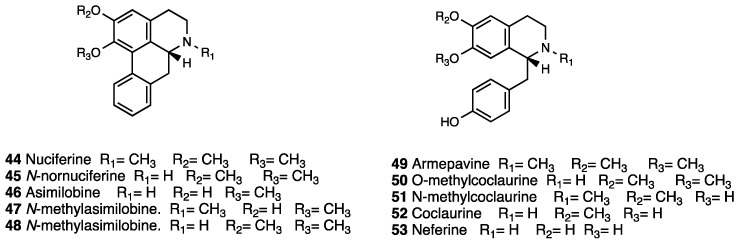
Benzyltetrahydroisoquinoline alkaloid structures (**44**–**53**) detected in the basic partition of *Nelumbo nucifera* ethanol extract.

Kumarihamy et al. [123] investigated the in vitro cannabinoid and opioid receptor binding affinities and the in vivo behavioral actions of *Nelumbo* flower extracts in order to isolate potential compounds that could treat CNS-associated disorders. White and pink flowers of *Nelumbo nucifera* were extracted with ethanol, followed by acid-base partitioning to yield benzyltetrahydroisoquinoline (BTIQ) alkaloids and long chain fatty acids, identified by physical and spectroscopic methods. A UHPLC/APCI-MS analysis of basic partitions revealed seven major peaks that matched with nuciferine (**44**), *N*-nornuciferine (**45**), asimilobine (**46**), *N*-methylasimilobine (**47**), *O*-methylasimilobine (**48**), armepavine (**49**), O-methylcoclaurine (**50**), *N*-methylcoclaurine (**51**), and coclaurine (**52**) (Figure 21) and three minor peaks, of which two matched with the dimeric bis-BTIQ alkaloids liensinine or isoliensinine and neferine (**53**, Figure 21); the third has not been identified. UHPLC/APCI-MS analysis of acid partitions revealed the presence of nuciferine (**44**) and *N*-nornuciferine (**45**) linoleic and palmitic acids, as well as stigmasterol.

Radioligand displacement and [^35^S] GTPγ binding assays demonstrated that ethanol extract, acid and basic partitions, and each isolated compound had no affinity for the CB-1 and CB-2 receptors. The acid and basic partitions, nuciferine (**44**), coclaurine (**52**), *O*-methylcoclaurine (**50**), and *N*-methylcoclaurine (**51**), were subjected to a tetrad assay, which is an indicator of classical cannabimimetic activity that induces hypomotility, catalepsy, hypothermia, and analgesia and is manifested by Δ^9^-THC and other cannabinoids. The in vivo mild cannabimimetic-type effect observed for the acid partition, as well as decreased locomotion and increased antinociception and hypothermia of the basic partition suggested the involvement of other CNS mechanisms. Moreover, while the acid partition was found inactive, basic partition and armepavine (**49**), *O*-methylcoclaurine (**50**), *N*-methylcoclaurine (**51**), coclaurine (**52**), and neferine (**53**) showed strong MOR and KOR displacement with the highest K_i_ values for *N*-methylcoclaurine (**48**, K_i_ = 0.9 μM), coclaurine (**49**, K_i_ = 2.2 μM), and *O*-methylcoclaurine (**47**, K_i_ = 3.5 μM). Neferine (**50**) displayed affinities for DOR and MOR with K_i_ values of 0.7 and 1.8 μM, respectively, and a weak DOR agonist (EC_50_ = 7.9 μM), weak MOR partial agonist (EC_50_ = 21 μM) profile. In Table 5, the affinity profiles of *Nelumbo nucifera* active principles are presented.

### 3.9. Clinacanthus nutans

**Figure 22:**
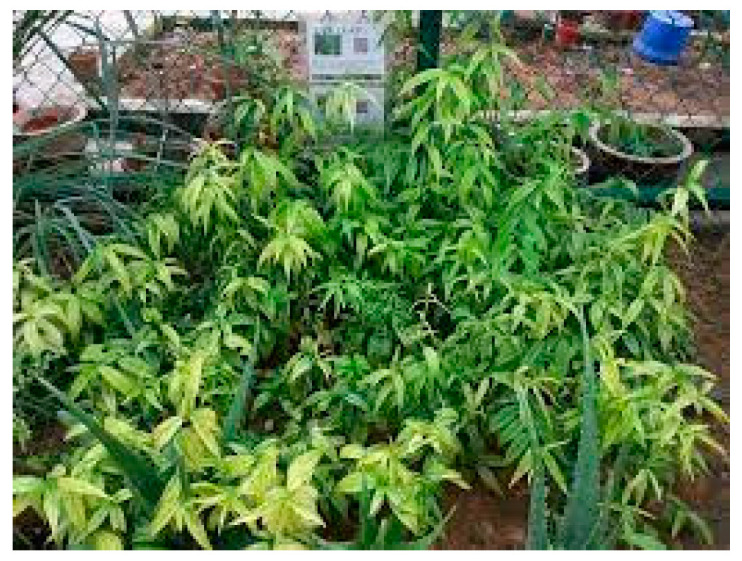
*Clinacanthus nutans* Lindau.

*Clinacanthus nutans* (Figure 22) Lindau, a plant belonging to the Acanthaceae family, known as “*Belalai Gajah*”, is a shrub native to tropical Southeast Asian countries.

Its stem and leaves are commonly used in folk medicine to treat several conditions such as rheumatism, hematoma, anemia, and menstrual pain, but they are also used against herpes simplex virus infections or snake bites. The methanolic extract of *Clinacanthus nutans* contains several flavonoids, as detected by UHPLC-ESI, that belong to the family of flavone C-glycoside, and the extract contains 16 phenolic compounds such as allic acid, 4-hydroxybenzoic acid, caffeic acid, coumaric acid, ferulic acid, schaftoside, vitexin, orientin, isoorientin, isovitexin, luteolin, apigenin, forsythosides H, forsythosides I, diosmetin glycoside, and diosmetin. Among them, at least gallic acid [124], caffeic acid [125], ferulic acid [126], vitexin [127], and apigenin [128] have been reported to exert antinociceptive activity. The attenuation of acetic acid-induced abdominal constriction, thermal response in hot plate tests, and reversion of the response latency in both phases of the formalin test suggested peripheral and central antinociceptive activity for the methanolic extract [129]. Pretreatment with naloxone blocked its effects in all three pain models, suggesting the involvement of opioid receptors. The lipid-soluble fraction of methanolic extract, obtained after its sequential extraction in a petroleum ether, was also investigated to determine the possible involvement of a non-opioid mechanism [130]. P.o.-tested, it showed the possible involvement of TRPV1-, NMDA-, B2-receptors, and a PKC-activated TRPV1 receptor pathway because of the petroleum ether extract’s capability to attenuate capsaicin-, glutamate-, PMA-, and bradykinin-induced nociception. This assumption was also supported by the partial reversion of petroleum ether antinociception by pretreatment with yohimbine, pindolol, caffeine haloperidol, and atropine, which are the antagonists of α2-adrenergic, β-adrenergic, adenosinergic, dopaminergic, or muscarinic receptors, respectively. Analogously, the methanolic extract of *Clinacanthus nutans* reversed, in a dose-dependent manner, the nociceptive effect of capsaicin-, glutamate-, phorbol 12-myristate 13-acetate-, and bradykinin-induced nociception models. This effect was inhibited by antagonists of MOR, DOR, KOR, α2-noradrenergic, β-adrenergic, adenosinergic, dopaminergic, and cholinergic receptors, as well as different K^+^ channels blockers [131].

### 3.10. Azadirachta indica Extracts and Oleum Azadirachti

**Figure 23:**
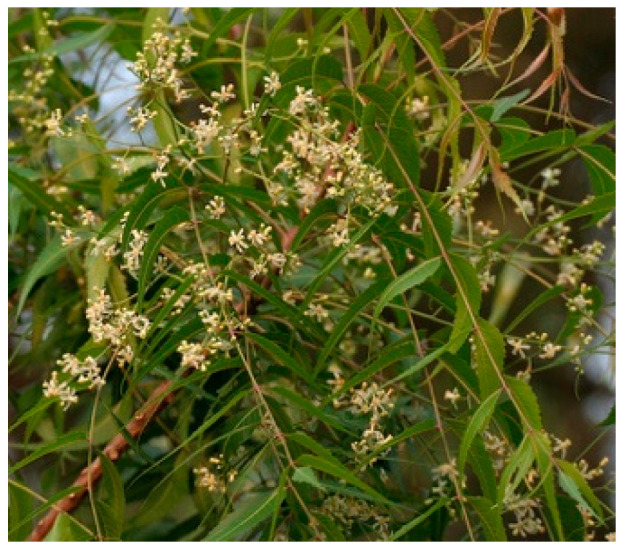
*Azadirachta indica* plant.

*Azadirachta indica* (Figure 23), a plant from Ayurveda, has been evaluated for a wide spectrum of diseases including cancer [132,133], inflammation [134], ulcers [135], immune disorders, hyperlipidemia [136], and liver disease [137].

**Figure 24:**
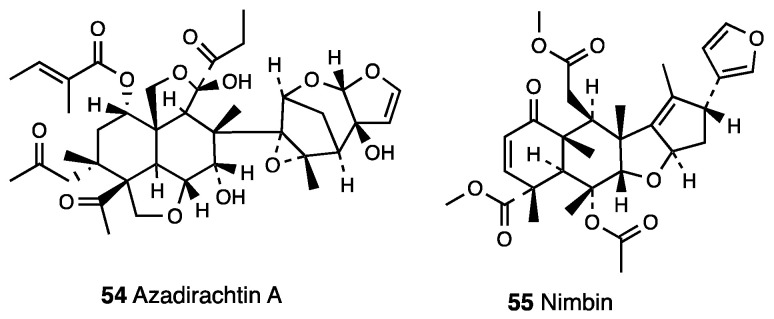
Major constituents (**54** and **55**) obtained from dried seeds oil of *Azadirachta indica*.

Kanagasanthosh et al. [138] also reported the antinociceptive effect of the ethanolic extract of *Azadirachta indica* leaves. Batista et al. [139], using a zebrafish model of pain, demonstrated the effectiveness of an *Azadirachta indica* fruit ethanolic extract in nociceptive pain. This extract produced a ketamine-reversed dose-dependent antinociceptive action on glutamate-induced nociception, indicating the possible modulatory action of the glutamatergic system. Moreover, the fruit extract decreased the nociceptive behavior in both phase I and II of a formalin test; this effect was completely reversed by naloxone, suggesting opioid receptor modulation. The ethanolic extract of the *Azadirachta indica* fruit also inhibited pain signs generated by the peripheral injection of acidic saline; its effect was reversed by amiloride (acid-sensitive ion channels antagonist) pre-treatment, indicating acid-sensitive ion channels as putative targets in this pain modulation. The potential capability of *Azadirachta indica* leaf extract to counteract typical behavior signs of neuropathic pain was also examined [140] by using a PSNL model. Further in vitro investigation established that the *Azadirachta indica* leaf extract attenuated increased levels of TNF-α, IL-1β, and NF-κB, as well as the mRNA expression of Bax, Caspase-3, and iNOs in neuropathic pain. The extract composition of several parts of the plant and the oil obtained from its dried seeds are different. Phenolic compounds and flavonoids are the major constituents of the extract, whereas oxidized tetranortriterpenes, including azadirachtin A (**54**, Figure 24), azadiriadione, epoxyazadiradione, azadirone, nimbidin, nimbin (**55**, Figure 24), deacetylnimbin, salannin, gedunin, mahmoodin, 17-hydroxydiradione, and related derivatives are the major constituents of the oil, also known as oleum azadirachti, obtained from its dried seeds.

Oleum azadirachti is widely used in folk medicine to treat many body disorders like gastric ulcers, cardiovascular disease, malaria, and rheumatism. Moreover, potential use of the oil as a contraceptive for intravaginal use, mosquito repellent, vaginal infections, gastric ulcers, cardiovascular disease, malaria, rheumatism and skin disorders, allergic skin reactions, asthma, bruises, colic, conjunctivitis, dysmenorrhoea, fever, gout, headache, itching due to varicella, kidney stones, psoriasis, and scabies has emerged from the literature [141]. Importantly, oleum azadirachti anti-inflammatory effects were also investigated. Nimbidin (40 mg/kg i.m.) reduced carrageenan-induced paw edema, formalin-induced arthritis in ankle joints, granuloma fluid exudation induced by croton oil, and granuloma induced by cotton pellets [142]. The major bioactive compound, Azadirachtin A (**54**, Figure 24), a compound belonging to the limonoid group, was found to inhibit the nociceptive responses of zymosan-induced writhing and hot plate tests in the extracts [143] at the dose of 120 mg/kg, and pre-treatment with the nonselective opioid antagonist, naltrexone (10 mg/kg, i.p.)—but not by a nonselective serotonergic antagonist, cyproheptadine—reversed its activity. Moreover, azadirachtin significantly reduced the acute carrageenan-induced paw edema and the proliferative phase of the inflammatory response, as demonstrated by the reduced formation of fibrovascular tissue induced by s.c. cotton pellet implantation [144].

### 3.11. Vitex megapotamica Extracts

**Figure 25:**
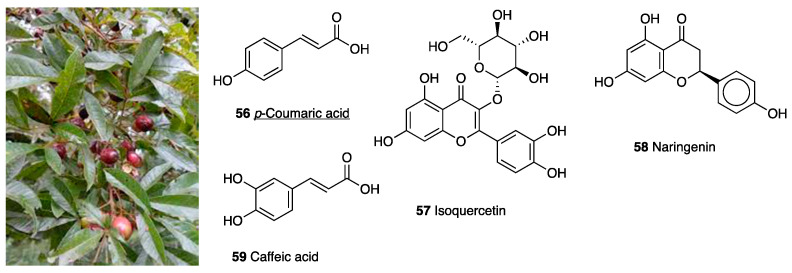
*Vitex megapotamica* and structures of primary active components (**56**–**59**).

Folk medicine reports *Vitex megapotamica* (Figure 25)—popularly known in Brazil as “tarumã”, a native tree from Brazil, Uruguay, Paraguay, and Argentina—leaf infusion to treat inflammatory diseases.

The UHPLC-ESI-MS/MS method identified phenolic compounds, especially *p*-coumaric acid (**56**, 9.3–61.8 mg g^−1^), isoquercitrin (**57**, 14.6–55.5 mg g^−1^), naringenin (**58**, 7.5–30.1 mg g^−1^), and caffeic acid (**59**, 12.7–18.3 mg g^−1^), as bioactive compounds in the extracts of *Vitex megapotamica* [145]. Obtained evidence suggested the crude extract functioned as an analgesic and antidepressant. Hamann et al. investigated the crude leaf extract in a p.o.-administered (10 mg/kg) Freund’s adjuvant-induced chronic inflammation and depression model [146], where it inhibited mechanical allodynia and thermal hyperalgesia without, however, reducing paw edema. In addition, the crude extract decreased immobility time in the forced swimming test. Both antinociception and antidepressant-like effects were prevented by naloxone. Moreover, the crude extract of the plant showed a safe profile; it did not alter locomotor activity and gastrointestinal function and, in a regimen of repeated administration, did not cause hyperalgesia.

### 3.12. (–)-Hardwickiic Acid

**Figure 26:**
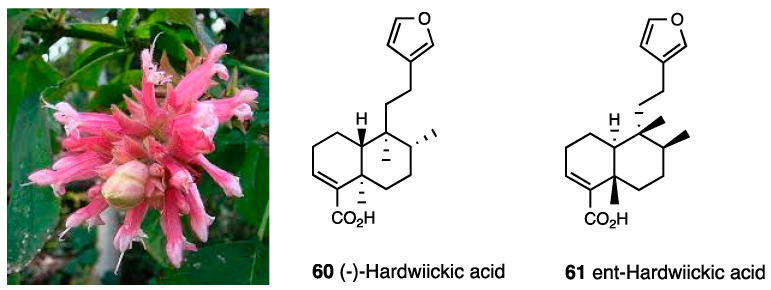
*Salvia wagneriana* and its active principles structures (**60** and **61**).

(–)-hardwickiic acid (**60**, Figure 26) is extracted from the aerial parts of *Salvia wagneriana* (Figure 26).

An i.t. (–)-hardwickiic acid injection (2 μg/5 μL) reversed mechanical allodynia on HIV- and paclitaxel-induced neuropathy through TTX-S voltage-gated sodium channels [147], starting from 1–2 h after the administration and lasting for 2–3 h. Moreover, it was shown to facilitate K^+^-evoked hippocampal NA but not dopamine, an effect prevented by NTI and nor-BNI, but not from the selective MOR antagonist CTAP, thus indicating (–)-hardwiickic acid action on a hippocampal NA overflow, evoked by mild depolarizing stimuli on presynaptic opioid receptor subtypes [148]. The (–)-hardwiickic acid enantiomer, ent-hardwiick acid (**61**, Figure 26), is found in the oleoresins of different species of *Copaifera*, popularly known as “copaíbas, copaibeiras, copaívas or oil stick”. Raw oleoresins have demonstrated traditional anti-inflammatory activity and are mostly composed of nonvolatile acid diterpenes and volatile sesquiterpenes [149]. A study conducted by Simaro et al. demonstrated that ent-hardwiickic acid inhibits the production of inflammatory cytokines that suppress the NF-κB pathway and exerts anti-inflammatory and analgesic effects in carrageenan-induced paw edema, formalin-induced pain, acetic-acid-induced abdominal writhing and tail flick tests.

### 3.13. Algrizea minor Essential Oil

**Figure 27:**
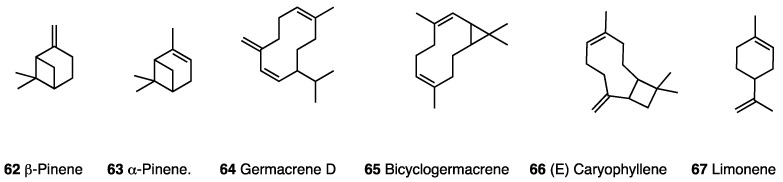
Structures of the major constituents (**62**–**67**) of the essential oil of *Algrizea minor*.

Recently, research identified essential oils produced by different species of the Myrtaceae family as potential candidates for the development of new drugs, with different antifungal, anti-bacterial, antiparasitic, and antinociceptive profiles [150]. In 2019, Olveira et al. developed a study with the aim of investigating the chemical composition, antimicrobial activity, antinociceptive activity, acute toxicity, and antioxidant activity of the essential oil of *Algrizea minor* (EOAm) [151]. It is composed of βpinene (**62**, 56.99%), αpinene (**63**, 16.57%), germacrene D (**64**, 4.67%), bicyclogermacrene (**65**, 4.66%), (E)-caryophyllene (**66** 3.76%), and limonene (**67**, 1.71%) (Figure 27).

It was demonstrated that the essential oil did not show acute toxicity in its maximum dose, and it was confirmed that it reduced pain with an opioid mechanism of action.

### 3.14. Verbascoside 

**Figure 28:**
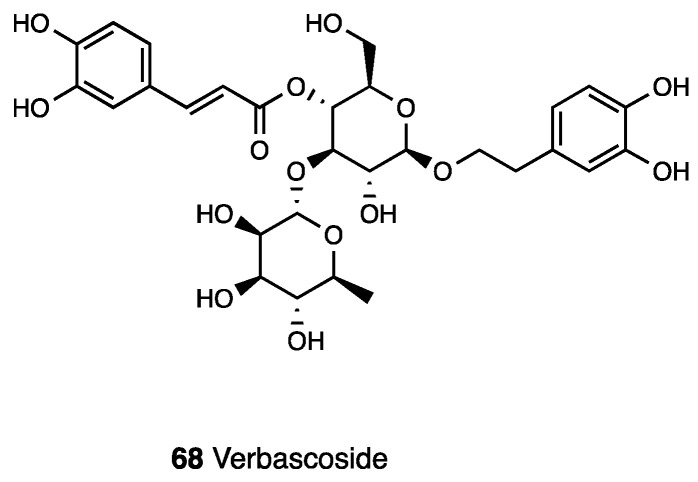
Verbascoside structure (**68**).

Verbascoside (**68**, acteoside, Figure 28) is a phenylpropanoid glycoside isolated from different medicinal plants that belong to various families such as Verbenaceae, Oleaceae, Buddlejaceae, Lamiaceae, Scrophulariaceae, and plants from traditional Chinese medicine.

The methanol and ethanol extracts of *Buddlejia globosa* from the Buddlejiaceae family did not show any differences in chemical compounds, among which verbascoside and 7-O-luteolin glycoside are the major chemical constituents. Verbascoside possesses extensive biological activity [152]: the anti-ulcerogenic and antispasmodic, immunomodulatory, antiproliferative, and inhibitory activity of telomerase. Moreover, it is known as an ROS-scavenging and antioxidant agent. Several studies confirmed its antinociceptive propriety after both i.p. and p.o. (300 mg/kg) administration [153,154,155] in a CCI model and in an intra-articular injection of sodium monoiodoacetate. The experiments in the CCI model showed a significant anti-hyperalgesic effect starting from 3 days and lasting up to 14 days post ligatures. It decreased cold allodynia and heat hyperalgesia. Verbascoside restored Bax/Bcl2 balancing, especially 3 dpl, and it reduced GFAP and Iba 1, 3, and 7 dpl; it increased GSH and MDA levels in the ipsilateral spinal cord of CCI animals, highlighting its immunomodulatory activity and antioxidant action. The data obtained reveal that the analgesic effect of verbascoside was comparable to that of gabapentin employed as a reference compound. Recently, Hara et al. demonstrated that its analgesic effect, when i.t.-administered, is mediated by MOR activation, because it was reverted only by naloxone and not by another antagonist such as atropine, bicuculline, or yohimbine. This finding was supported by previous studies that indicated a binding profile vs. MOR for this compound [156]. Indeed, verbascoside dose-dependently displaced DAMGO and was able to directly interact with MOR. Moreover, the effects of caffeic acid and hydroxytyrosol on neuropathic pain were studied to determine the verbascoside component that mediates its antihyperalgesic effects. They were administered via i.t., and results showed that caffeic acid suppressed hyperalgesia only at a high dose—an effect that was not affected by naloxone while hydroxytyrosol possessed no effect. Verbascoside has also anti-inflammatory and antinociceptive activities as was demonstrated in carrageenan-induced hind paw edema and p-benzoquinone-induced abdominal constriction. On the other hand, to overcome verbascoside limits in clinical application due to its poor chemical stability depending on pH, Isacchi et al. optimized an unilamellar liposomal formulation of verbascoside (100 mg/kg), demonstrating a significant persistent anti-hyperalgesic activity in paw pressure tests in CCI animals when compared to a same dose of a free drug. Interestingly, the main difference was recorded starting from 45 min after administration [157].

**Figure 29:**
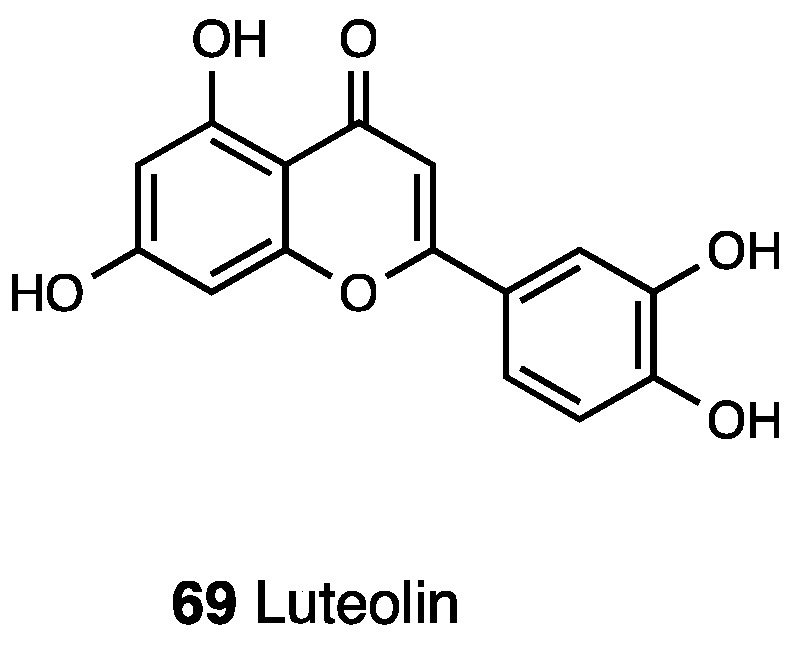
Luteolin structure (**69**).

Luteolin, which chemically belongs to flavonoids (**69**, Figure 29), is largely found in many parts such as the leaves, bark, and seeds of several plants; indeed, it is concentrated in fruits and vegetables present in a human diet.

Luteolin has different proprieties like anticancer, anti-diabetic, and antioxidant effects, and these include the inhibition of iNOS expression and activity and reduction of TNF-α, IL-6 mRNA, and protein levels, in addition to analgesic and anti-inflammatory activity through NF-kB signaling inhibition, as showed by several studies [158,159,160,161]. Hara et al. reported the anti-hyperalgesic effect of luteolin, i.t.-injected at a dose of 1.5 μg in mechanical, lower in thermal, and especially in heat hyperalgesia. Interestingly, i.t. pretreatment with naloxone and bicuculline significantly inhibited the effect of luteolin. On the other hand, their experiments displayed the fact that i.c.v. injection did not show the same effect, indicating a different action between supraspinal and spinal levels [158]. Anti-inflammatory activity for luteolin’s derivates was also reported; for example, luteolin-5-O-glycoside can decrease COX-2 [162], while luteolin-7-O-glycoside inhibits leukotriene C4 production in macrophages [163].

### 3.15. Aqueous Extracts of Stachytarpheta cayennensis

**Figure 30:**
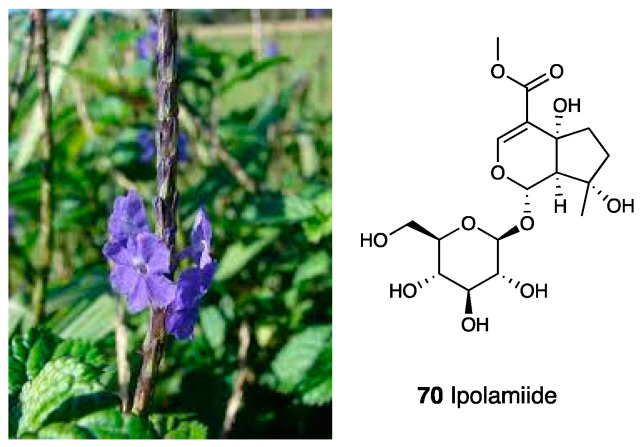
*Stachytarpheta cayennensis* and its active compound structure (**70**).

*Stachytarpheta cayennensis* (Figure 30) (L.C. Rich.) Vahl (Verbenaceae) is a purple flower diffused mainly in Brazil. It is used in Brazilian folk medicine for its anti-inflammatory, analgesic, antipyretic, hepatoprotective, and laxative properties. In the literature, it is reported that freeze-dried aqueous extracts (AEs) obtained from entire or selected parts of the plant were tested for their effects on inflammation and pain in rodents.

AE-Total significantly reduced abdominal writhing induced by acetic acid without altering the writhes induced by acetylcholine. AE-Total did not show analgesic effects in different models of pain such as formalin and capsaicin or tail flick tests [164]. To evaluate the alcoholic and *n*-butanolic extracts of the dried leaves of *Stachytarpheta cayennensis* in anti-inflammatory and antinociceptive models, the fraction (F5), characterized by the presence of the iridoid glycoside ipolamiide (**70**),] and the phenylethanoid glycoside acteoside o verbascoside (**68**), showed the highest anti-inflammatory activity-inhibiting chemical mediators of the inflammatory process, as well as the histamine and bradykinin-induced contractions of GPI. These glycosides, via p.o. administration, also demonstrated positive responses vs. edema formation, demonstrating that the anti-inflammatory effect of *Stachytarpheta cayennensis* was not due to an irritating action. All the assayed extracts also showed an antinociceptive activity in the hot plate test [165].

### 3.16. Conolidine 

**Figure 31:**
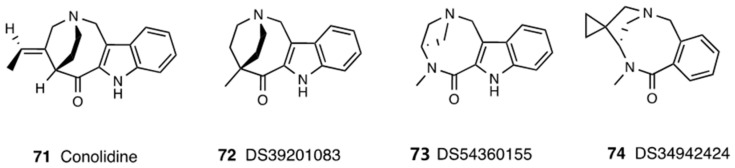
Structures of *Tabernaemonta divaricate* active principles (**71**–**74**).

Conolidine (**71**, Figure 31) isolated from the bark of the flowering shrub *Tabernaemonta divaricata* is mainly employed in traditional Chinese and Thai medicine for pain and fever treatment. It contains different alkaloids that possess a carbon-based framework similar to opioids.

Conolidine is able to induce analgesia, and it does not cause the adverse effects of classic opioid drugs. Experimental studies highlighted that its effects are due to the chemokine receptor (ACK3), a broad-spectrum scavenger of opioid peptides and the dynorphin, enkephalin, and nociceptin families [166]. Recently, ACK3 modulation has been proposed as a different opioid system target, and studies carried out by Szpakowska et al. reported that it could be highly responsive to conolidine. Therefore, conolidine is defined as a potent non-opioid compound lacking opioid analgesic side effects such as nausea, respiratory depression, constipation, tolerance, and physical dependence [167]. In recent years, different varieties of conolidine derivatives were synthesized. In particular, a novel compound, 5-methyl-1,4,5,7-tetrahydro-2,5-ethanoazocino[4,3-b]indol-6(3H)-one sulfuric acid salt (DS39201083, **72**, Figure 31), was obtained. It possesses a unique bicyclic skeleton and shows a more potent analgesic effect than conolidine in an acetic acid-induced writhing test and formalin test in ddY mice but did not show any agonist activity at MOR [168]. Similarly, another conolidine derivative, (5S)-6-methyl-1,3,4,5,6,8-hexahydro-7H-2,5-methano[1,5]diazonino[7,8-b]indol-7-one sulfate salt (DS54360155, **73**, Figure 31), was discovered. It had a unique and bicyclic skeleton and was an analgesic more potent than conolidine, but it did not exhibit MOR agonist activity. Recently, a novel compound, (5′S)-10′-fluoro-6′-methyl-5′,6′-dihydro-3′H-spiro[cyclopropane-1,4′-[2,6]diaza[2,5]methano[2,6]benzodiazonin]-7′(1′H)-one, was identified and named DS34942424 (**74**, Figure 31). It was characterized by a simple benzene scaffold and the original bicyclic structure consisting of an amide bond and a pyrrolidine ring. This compound showed potent analgesic efficacy after p.o. administration in mice, as revealed by both the acetic acid-induced writhing test and the formalin test. It had a good safety profile, but its mechanism of action is still unknown.

### 3.17. Rubiscolin

**Figure 32:**
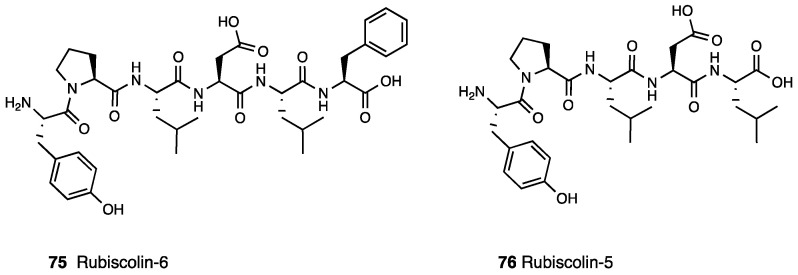
Structures of peptides derived from D-ribulose-1,5-bisphosphate carboxylase/oxygenase (**75** and **76**).

Rubiscolin is a bio-active peptide derived from the D-ribulose-1,5-bisphosphate carboxylase/oxygenase (RuBIsCO), which is the major protein responsible for carbon dioxide fixation and photorespiration in the green leaves of plants. In recent years, rubiscolin-6 (**75**, YPLDLF, Figure 32) and rubiscolin-5 (**76**, YPLDL, Figure 32) have been identified as bioactive peptides derived via digestion of RuBIsCO from spinach leaves.

Differently from opioid peptides that have aromatic amino acids in the third position and are MOR selective, rubiscolins structurally characterized by an aliphatic amino acid in the third position are DOR selective and exhibited an antinociceptive effect in mice even after p.o. administration [169]. Recently, the SAR of rubiscolin analogues were studied by using 3D- QSAR analysis, and traditional CoMFA and CoMSIA approaches have been employed to derive quantitative relationships between the structure of rubiscolin analogues and their activity towards DOR. This model suggested that the DOR activity of rubiscolin analogues exhibited a relevant relationship with the local hydrophobic and hydrophilic characteristics of amino acids at positions 3, 4, 5, and 6. Other studies highlighted that rubiscolin-6 is more potent and has a stronger affinity for DOR than rubiscolin-5. Rubiscolin peptides are characterized by oral bioavailability, and in particular, rubiscolin-6 produced analgesia in a tail pinch assay with few side effects [170]. The opioid activities of those derivatives were tested by MVD and GPI assays. The results displayed that their effects in MVD and GPI assays were reversed by naloxone, confirming opioid receptor involvement [171].

### 3.18. Tingenone

**Figure 33:**
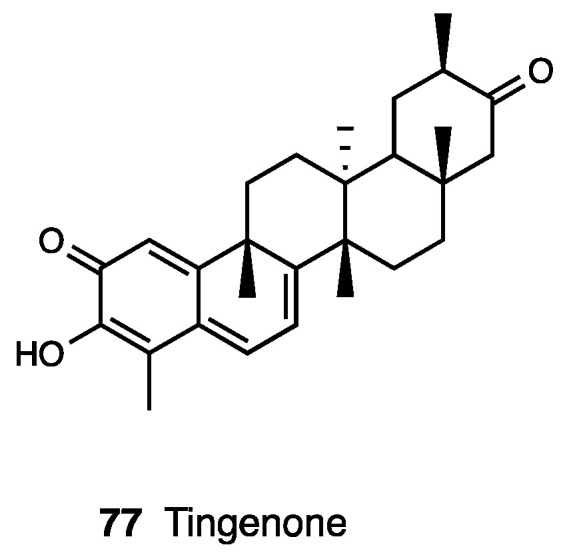
Tingenone structure (**77**).

The celastraceae family, which comprise 1220 species growing in tropical regions of the world, was used in folk medicine to treat several inflammatory diseases, such as gastric disorders, fever, and cancer. In particular, the hexane and ethyl acetate extract of *Maytenus ilicifolia* showed an antinociceptive effect, while the chloroform extract of *Maytenus senegalensis* displayed anti-edema action [172,173,174]. Several types of the root extract of *Maytenus imbricata* showed antinociceptive effects, but only the hexane/ethyl ether extract was able to reduce licking time in both phases of the formalin test and edema formation in the carrageenan paw test, also indicating an anti-inflammatory action, probably through prostaglandin synthesis inhibition. The antinociceptive effect of tingenone, a pentacyclic triterpene (**77**, Figure 33) isolated from *Maytenus imbricata* roots, was also investigated.

Veloso et al. demonstrated that the opioid system is involved in tingenone’s peripheral antinociceptive activity but not directly via opioid receptors—rather, through endogenous opioid peptide release by immune cells. Moreover, it was found that tingenone’s peripheral analgesic activity was also related to CB_2_ receptor activation [175,176]. Both systems act through the L-arginine/NO/cGMP/K^+^ATP pathway, as demonstrated via treatments with several inhibitors.

### 3.19. Esters of N-Methylanthranilic Acid

**Figure 34:**
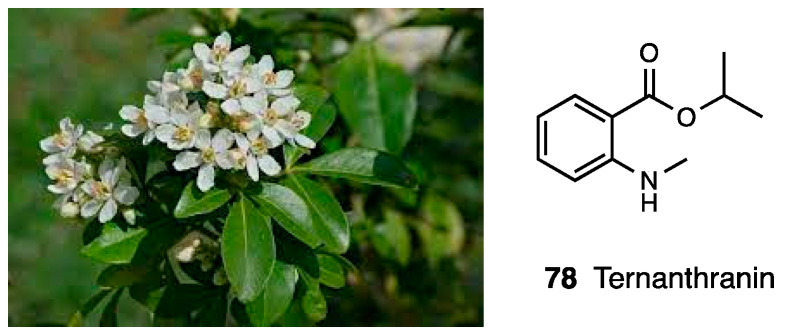
*Choisya ternata* and ternanthranin structure (**78**).

Esters of *N*-methylanthranilic acid are chemical compounds present in several plants with an important role in plant–plant or plant–insect interactions. These are the skimmianine, or evoxine alkaloids, which are used as sedatives and spasmolytics. *Choisya ternata* Kunt, also called “Mexican orange” is a very popular plant in Mexico and North America, which emits a pungent smell when its leaves are brushed (Figure 34).

Using fresh and air-dried plants, Radulovic et al. obtained and characterized two essential oils, the second containing a high concentration of sesquiterpenoids. They focused their attention on methyl- and isopropyl-*N*-methylanthranilates, sometimes difficult to detect. To test their antinociceptive effect, they synthetized methyl-, propyl-, and isopropyl *N*-methylanthranilates and evaluated both in an acetic acid-induced writhing model and in the hot plate test, comparing them with the essential oil and ethanol extract of *Choisya ternata* Kunt. Their results indicated that in both models, substitution played a pivotal role; indeed, the methyl derivate showed the least activity, whereas the essential oil and ethanol extract exhibited about a hundred times less activity [177]. To deepen the antinociceptive mechanism of these derivates, they assayed isopropyl, propyl, and methyl *N*-methylanthranilates in capsaicin and glutamate-induced nociception via formalin and hot plate tests after oral administration at doses between 0.3 and 3 mg/kg. The compounds significantly reduced the time animals spent licking in the second phase of the formalin test, while in the first phase, this was only achieved with the highest dose (30 mg/kg). In capsaicin and glutamate-induced nociception, they produced a significant inhibition of the nociceptive response, indicating the involvement of vanilloid and glutamate receptors. The results from the hot plate test also showed a supraspinal antinociceptive effect for all three derivates, which was reverted by naloxone and yohimbine pre-treatment, confirming a synergic activity between opioid and adrenergic systems in controlling pain [178,179]. These results were corroborated by another study in which the same authors demonstrated the anti-inflammatory effect of essential oil from *Choisya ternata* Kunt and isopropyl- (also known ternanthranin, **78**), methyl-, and propryl-*N*-methylanthranilate in an SAP model and the reduction of leukocyte migration, protein leakage, and the level of several pro-inflammatory cytokines such as NO, IL-1β, and TNF-α. Interestingly, they confirmed the antiedematogenic action of ternanthranin and methyl- and propyl-*N*-methylanthranilate trough direct interaction with serotonin, bradykinin and prostaglandin receptors [180].

### 3.20. Mansoa alliacea Extract

**Figure 35:**
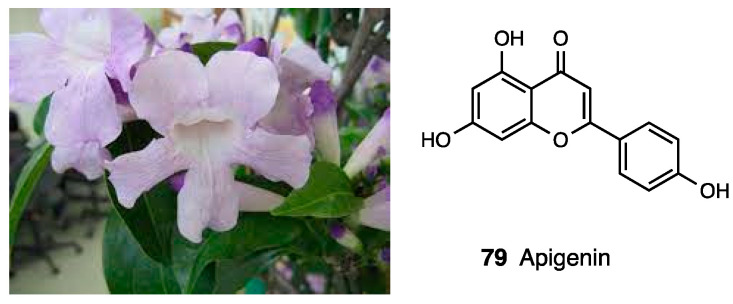
*Mansoa alliacea* and apigenin structure (**79**).

*Mansoa alliacea* (Figure 35), also known as “false garlic” because of the smell produced by its crushed leaves, is a typical plant of Southern Mexico used in traditional medicine as an infusion to treat fever and rheumatic pain. Despite its common use, there are limited studies focusing on its analgesic activity. Valle-Dorado et al. evaluated the ethanolic and aqueous extract of *Mansoa alliacea* in both formalin and hot plate tests, comparing both i.p. (30, 100 and 300 mg/kg) and oral (300 mg/kg) administration. Generally, both extracts showed a dose-dependent antinociceptive effect, but ethanol extract was more efficacious in both phases of the formalin test. Moreover, in the hot plate test, only the ethanolic extract at the dose of 300 mg/kg i.p. significantly increased latency, starting from 60 min, while the aqueous extract showed the same effect at 600 mg/kg. Furthermore, only the antinociceptive action of the ethanol extract was inhibited by naloxone pre-treatment in both phases of the formalin test, in agreement with previous studies that confirmed an opioid binding profile, especially for DOR, by apigenin (**79**, Figure 35)—the major constituent of this extract. This confirms that different concentrations and types of constituents, depending on the polar nature of extracts, can influence the antinociceptive mechanism of action [181]. These results were confirmed in the studies of Hamman et al., which evaluated the antinociceptive effect of hydroethanolic extract in CFA-induced mechanical allodynia through a tail flick test [182].

### 3.21. Cardamonin

**Figure 36:**
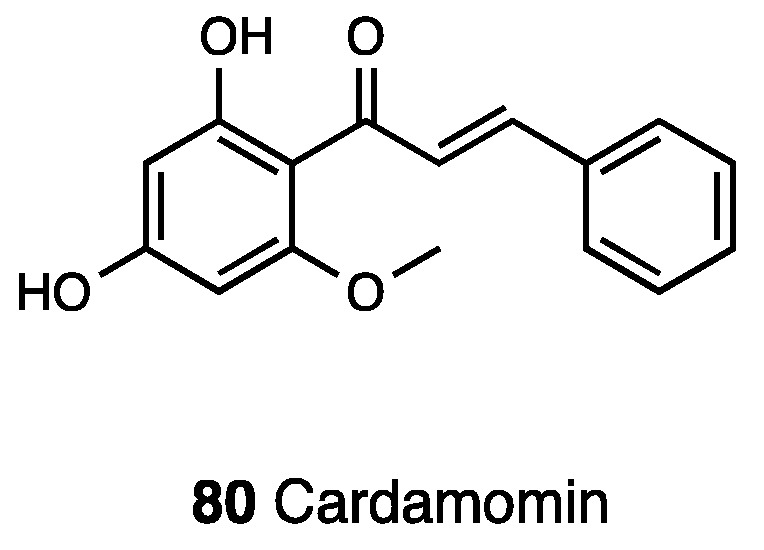
Structure of 2′,4′-dihydroxy-6′-methoxychalcone (**80**).

Cardamonin, or 2′,4′-dihydroxy-6′-methoxychalcone (**80**, Figure 36), is a naturally occurring chalcone isolated from the seeds of several plant species such as *Amomum subulatum*, *Boesenbergia pandurata*, *Alpinia rafflesiana*, *Alpinia katsumadai*, *Alpinia henryi*, and *Campomanesia adamantium*.

Literature data reported a sequela of biological activity for Cardamonin [183,184,185,186,187,188,189,190] As is common for phenolic compounds, it in vitro inhibits NO and PGE2 expression via interruption of the NF-κB pathway [191,192]. The capability of Cardamonin to suppress the expression of COX-2 and transglutaminase-2, PBQ-induced writhing, and carrageenan-induced hyperalgesia has also been demonstrated [193]. Cardamonin exerts significant peripheral and central antinociception in chemical- and thermal-induced nociception in mice, as assessed by acid acetic–induced abdominal writhing, formalin, and hot plate tests (i.p.- and p.o.-administered at the range doses of 0.3–10 mg/kg) [194]. The absence of a myorelaxant or sedative effect in the antinociception effect was verified through a rotarod assay. The involvement of TRPV1 in cardamonin antinociceptive activity was demonstrated by using a capsaicin-induced paw licking model, where the compound was effective at the dose of 3 mg/kg. In vitro investigations found cardamonin to be a selective blocker of TRPA1 but not of TRPV1 nor TRPV4 channels [195]. At all dosages, cardamonin produced significant antinociceptive activities in glutamate-induced nociception. The effect of cardamonin in phase I of the formalin test was reversed by pre-treatment with naloxone, indicating the involvement of opioid receptors. Analogous evidence emerged in a study conducted by Sambasevam et al. [196], which showed cardemonin’s (3–30 mg/kg) anti-hyperalgesic and anti-allodynic effects reversed by pre-treatment with naloxone and naloxone methiodide in a CCI model of neuropathic pain. The serotonergic pathway seems to play a central role in cardamonin anti-neuropathic effects through the involvement of the 5-HT1A receptor subtype. Indeed, the administration for four consecutive days before cardamonin treatment of the inhibitor of serotonin synthesis, *ρ*-chlorophenylalanine, reversed cardamonin-induced antihyperalgesic and antiallodynic effects Moreover, mice pretreatment with several 5-HT receptor subtype antagonists abolished the anti-neuropathic effects of the compound, which also showed the capability to upregulate 5-HT1A expression in the brainstem and spinal cord [197].

### 3.22. Berberine 

**Figure 37:**
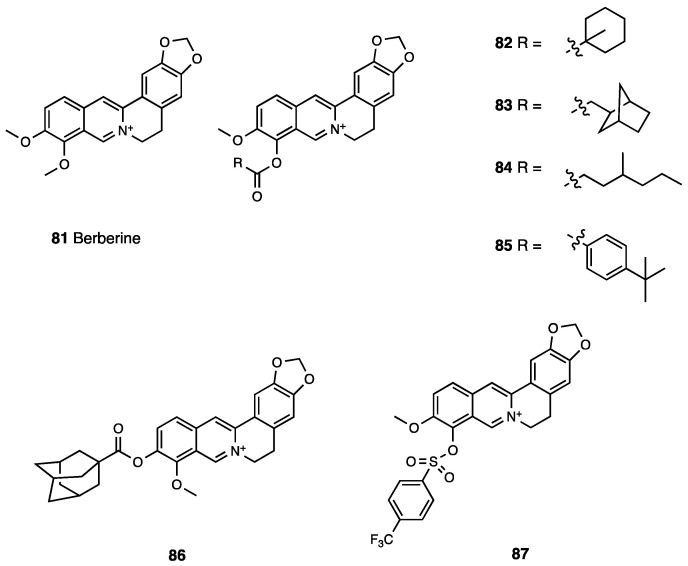
Structures of berberine and its semisynthetic derivatives (**81**–**87**).

Berberine (**81**, Figure 37) is an isoquinoline alkaloid present in the *Coptis* and *Berberis* species and features a variety of properties such as anti-oxidant [198], anti-tumor, anti-bacterial [199], hepatoprotective [200], anti-cholinesterase [201], anti-inflammatory [202], and pain-relieving [203] effects.

Berberine is effective in different pain models [204]. In a rat model of STZ-induced diabetic neuropathy, berberine anti-allodynic and anti-hyperalgesic effects were related to the increased expression of MOR mRNA coupled with decreases of ROS, TNF-α, IL-6, and SOD levels [205]. Berberine pain-relieving activity was exerted primarily via down-regulation of TRPV-1 and suppression of NF-κB. Anti-allodynic and anti-hyperalgesic effects shown by berberine in chemotherapy-induced neuropathic pain were mediated by Nrf2 gene expression increase. In SNL neuropathic pain, berberine antinociceptive effects, coupled with its low tolerance inducing capability, were related to TRPV1 expression decrease in dorsal root ganglion neurons [206]. Berberine, via MOR and DOR modulation, alleviates murine models of visceral pain, increasing protein receptor expression [207,208]. Berberine’s repression of inflammatory markers was related to its capability to reduce inflammatory chronic pain [209,210]. Several papers report its anti-cholinesterase activity as a putative additional analgesic mechanism [211,212]. Despite its fascinating pharmacological fingerprint, berberine therapeutic use is compromised by its unfavorable pharmacokinetic properties. Indeed, the quaternary ammonium cation in berberine structure confers to alkaloid low water solubility, reflecting poor absorption and bioavailability. Thus, to improve berberine therapeutic potential, several modifications were performed in its nucleus to obtain derivatives with improved pharmacodynamic and pharmacokinetic profiles [213]. Wang et al. synthesized berberine derivatives by modifying the substituents of the D ring. Newly synthesized derivatives were evaluated for their capability to suppress TNF-α-induced NF-κB activation. Emerging from SAR studies, tertiary/quaternary carbon substitutions at position 9 or rigid fragments at position 10 enhanced its anti-inflammatory potency. Among them, compounds **82**–**85**, **86**, and **87** (Figure 37) were the most potent, whereas compounds with amino or amido groups were less potent than berberine [214].

**Figure 38:**
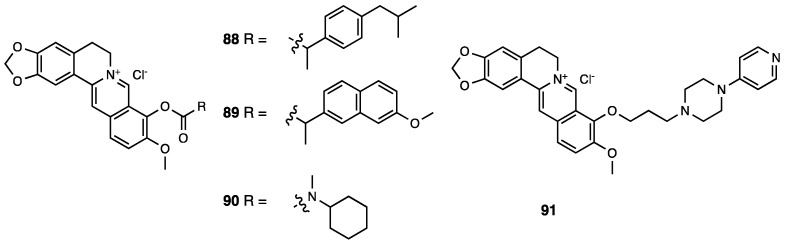
Structures of 9-*O*-modified derivatives of berberine (**88**–**91**).

To evaluate the influence of the carboxylic group in berberine-induced anti-inflammatory effects, 9-O-substituted derivatives (Figure 38) were synthesized and evaluated in vivo using a xylene-induced animal pain model [215]. Higher anti-inflammatory effects, in comparison to berberine, were reported for **88** and **89** (Figure 38) where the carboxylic group was esterified with ibuprofen and naproxen, respectively. Compounds **88** and **89**, similarly to berberine, reduced TNF-α and IL-6 levels in serum. Other 9-O-modified berberine derivatives were synthesized and in vitro and in vivo evaluated by Huang et al. [216]. These compounds significantly inhibited NO release and reduced IL-6 and TNF-α level production in transgenic zebrafish larvae injuries. Moreover, **90** and **91** (Figure 38) were able to reduce the migration of primitive macrophages and neutrophils in in vivo models of zebrafish larvae.

### 3.23. Lappaconitine

**Figure 39:**
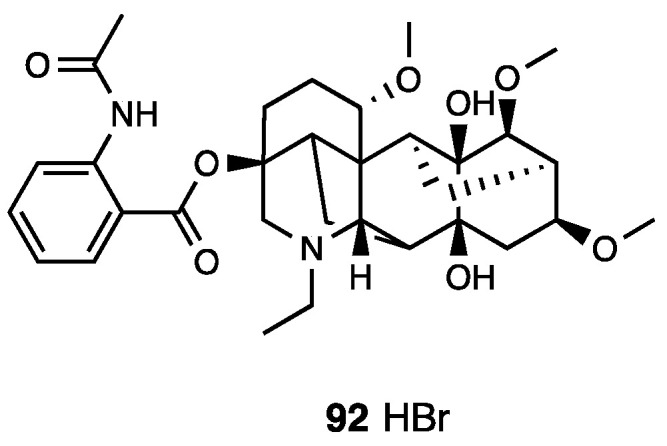
Structure of Lappaconitine Hydrobromide (**92**).

Lappaconitine (LA, **92**, Figure 39) is a monoester diterpene alkaloid extracted from the root of a natural plant belonging to the *Aconitum* species [216]. It has been clinically employed for the treatment of different types of pain such as cancer pain, post-surgical, and sciatic pain because of its analgesic effects. Due to its poor solubility, it is used via p.o. and i.v. administration in the form of Lappaconitine Hydrobromide (LAH, **92**·HBr, Figure 39), obtained by LA reacting with hydrobromic acid. Because the presence of fluorinated substituents in the process of drug design are able to improve drug absorption, Lappaconitine trifluoroacetate (LAF), with an increased fat solubility, was synthesized by introducing an organofluorine group to LA [217].

Literature studies report that this new compound had a lower toxicity and was demonstrated to have an improved analgesic effect and a longer half-life in comparison with LAH. Moreover, in a recent study, it was reported that the in vitro transdermal permeation of LAF was higher than LAH, indicating that LAF can be conveniently used for transdermal drug delivery (TDD). It was observed that LA, administered either systemically or via i.t, attenuated mechanical allodynia and thermal hyperalgesia in neuropathic and bone cancer pain. LA induced dynorphin A expression in cultured primary microglia and in the spinal cord of neuropathic rats, and its antinociceptive effect was completely blocked by an i.t. injection of the specific dynorphin A antibody and KOR antagonist. Therefore, these results suggest that LA produces antinociception through the stimulation of spinal microglial dynorphin A expression [218]. Recently, it was demonstrated that LA exerted an inhibitory effect on the nociceptive behaviors induced by CCI, decreasing the expression of the P2X3 receptors in the dorsal root ganglion neurons of CCI rats [219]. LA effects were also evaluated by the writhing response to acetic acid and formalin and hot plate tests. Obtained data revealed that LA possessed a notable central dose-dependent analgesic effect, and that it could significantly suppress egg albumen-induced paw edema in rats and xylene-induced ear swelling in mice, showing a good anti-inflammatory effect [220,221].

### 3.24. Oleanolic Acid

**Figure 40:**
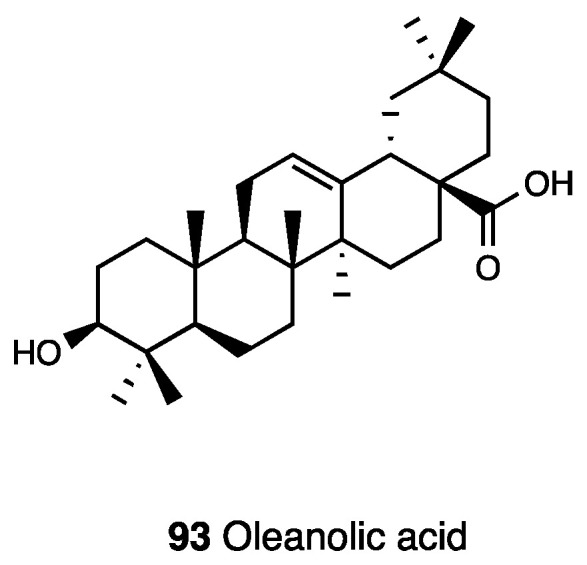
Oleanolic acid structure (**93**).

Oleanolic acid is a pentacyclic triterpenoid compound (**93**, Figure 40), found as a free acid or as an aglycone with its isomer, ursolic acid, in more of 1620 medicinal plants—especially in their fruits, such as *Lantana camara* and *Lisgustrum lucidum*—but also in *Rosmarinus officinalis* and in other species of the Lamiaceae family.

This compound was widely used for different chronic diseases such as cancer, liver injury, atherosclerosis, and inflammation thanks to its potent effects, which caught the interest of several researchers. Maia L.J. et al. investigated oleanolic acid extract from aerial parts of *Eriope blanchetii* (Lamiaceae) in a model of visceral pain induced by an intracolonic instillation of mustard oil, which is an algogenic substance that induces both pain-related behavior and inflammatory reactions in mice. They found that p.o. oleanolic acid (3, 10 and 30 mg/kg) demonstrated an antinociceptive effect, reversed only by naloxone pretreatment, indicating an opioid involvement in its mechanism. Moreover, in treated mice, oleanolic acid did not affect locomotion; rather, it reverted mustard oil-induced hypolocomotion [222]. Opioid involvement in oleanolic analgesic effects was also confirmed by Park S. H. et al., who also demonstrated its serotoninergic—but non-adrenergic—involvement in its antinociceptive action in various pain models like acetic acid-induced writhing and formalin tests [223]. Recently, Li X. et al. evaluated the analgesic effect of oleanolic acid—i.p. administered—in a model of SNL neuropathic pain in a dose ranging from 2 to 10 mg/kg. Oleanolic acid dose-dependently showed an antihyperalgesic effect both in mechanical and thermal hyperalgesia. Interestingly, they discovered that oleanolic acid restored altered M1/M2 microglia polarization, decreased IL-6, TNF-α and IL-1β levels, and increased IL-10 levels. In addition, it was able to suppress inflammatory responses in LPS-treated microglia by blocking the TRL4-NF-kB pathway, corroborating its anti-inflammatory action [224]. Despite its properties, low water solubility and instability slow its development as a pharmaceutical product. For this purpose, Rali et al. tried to improve its bioavailability by synthetizing some acetate and ester derivates. They demonstrated that, in a tail flick test, trifluoroacetyl derivates did not display a better analgesic profile compared to oleanolic acid, which showed an earlier onset of action. On the other hand, in the first phase of a formalin test, only the 28-methylester and 3-acetyloleanane derivate significantly increased pain thresholds, while in the second phase, only trifluoroacetyl derivates showed a significantly better analgesic activity than oleanolic acid. In albumin-induced inflammation, trifluoroacetyl derivates showed anti-inflammatory effects that were better than other compounds, indicating that the trifluoroacetyl group can influence oleanolic acid proprieties [225].

### 3.25. Urundeuvine A, B and C

**Figure 41:**
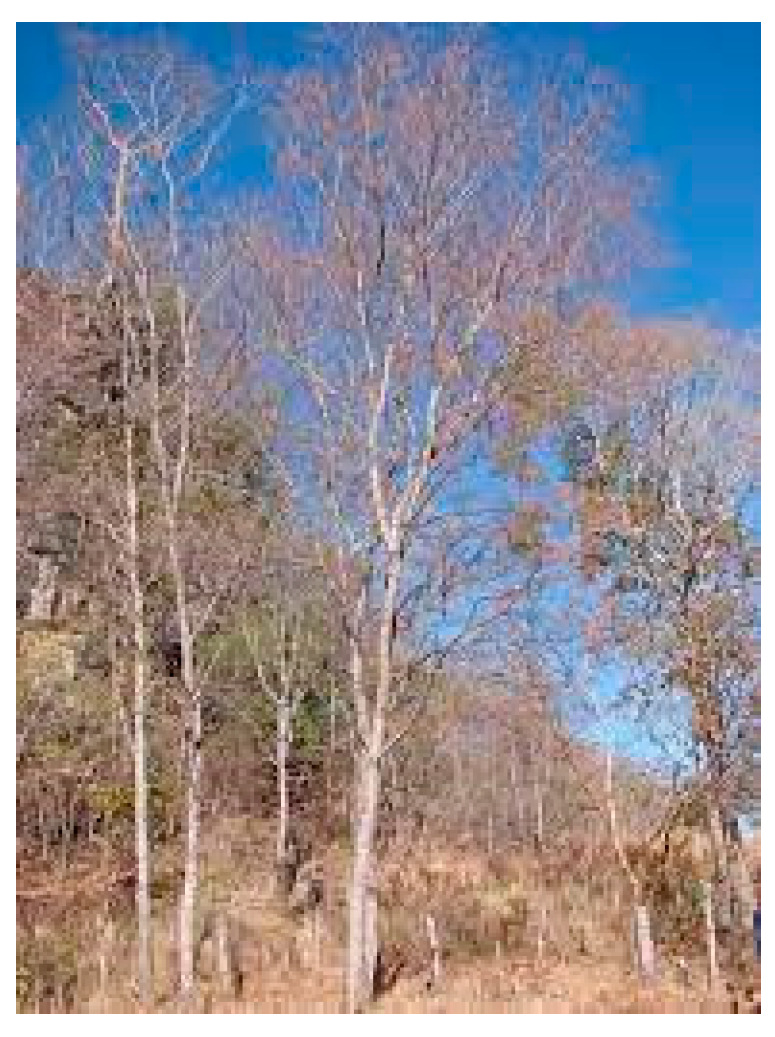
*Myracrodruon urundeuva* Allemao.

*Myracrodruon urundeuva* Allemao, belonging to the Anacardiaceae family, is a tree common in several regions of Brazil, especially in the North-East region (Figure 41), and is used in popular medicine to treat female genital tract inflammation, respiratory diseases, and wounds in the skin.

**Figure 42:**
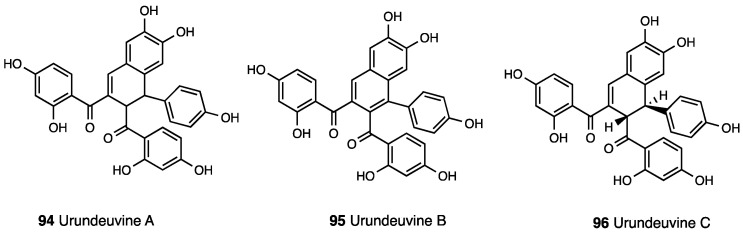
Structures of urundeuvine A, B and C (**94**–**96**).

Ethyl acetate extract contains up to seven different chemical compounds—chalcone- and tannin-enriched fractions showing interesting pharmacological activity in pain conditions, among these. Chalcones are chemical compounds belonging to the flavonoid class and are widely used for their antioxidant and anti-inflammatory activities, but they are also used to obtain stronger capillary walls and prevent bleeding. In the *Myracrodruon urundeuva* extract, there are three dimeric chalcones: urundeuvine A, B, and C (**94**–**96**, Figure 42).

Viana et al. first demonstrated the antinociceptive effect of chalcone-enriched fractions obtained from the ethyl acetate extraction of *Myracrodruon urundeuva* stem bark in several pharmacological tests, which were evaluated in doses ranging from 5 to 20 mg/kg and administered via both i.p. and p.o. In acid-acetic induced writhing, the formalin test, and hot plate test, they found a better analgesic activity for i.p. with respect to oral administration, which demonstrated irregular absorption. In the formalin test, the extract especially reduced pain behavior in the second phase, and it was partially reverted via naloxone pretreatment, while in the hot plate test, a significant increase of latency was observed for a dose of 10 and 20 mg/kg i.p. after 30 and 60 min. Furthermore, they found a significant anti-edematogenic activity for 40 mg/kg, administered via both i.p. and oral administration, after 3 and 4 h from a carrageenan injection [226]. Tannins, like flavonoids, are polyphenol compounds produced by plants as protective agents and can be found condensed with protein or other macromolecules like cellulose and minerals. Holding a polyphenol structure, they have a strong antioxidant activity. Considering the analgesic action, Souza S.M.C. et al. tested tannin-enriched fractions at doses of 5, 10, and 50 mg/kg i.p. in a formalin test, showing a dose-dependent antinociceptive action predominately in the second test phase, which was not reverted by naloxone pretreatment, indicating a non-opioid analgesic action for this extract. Moreover, they also demonstrated a significant anti-inflammatory action for an i.p.-administered dose of 10 mg/kg, starting from 3 h after carrageenan injection, which became less evident after p.o. administration [227].

### 3.26. Artemisia annua L.

**Figure 43:**
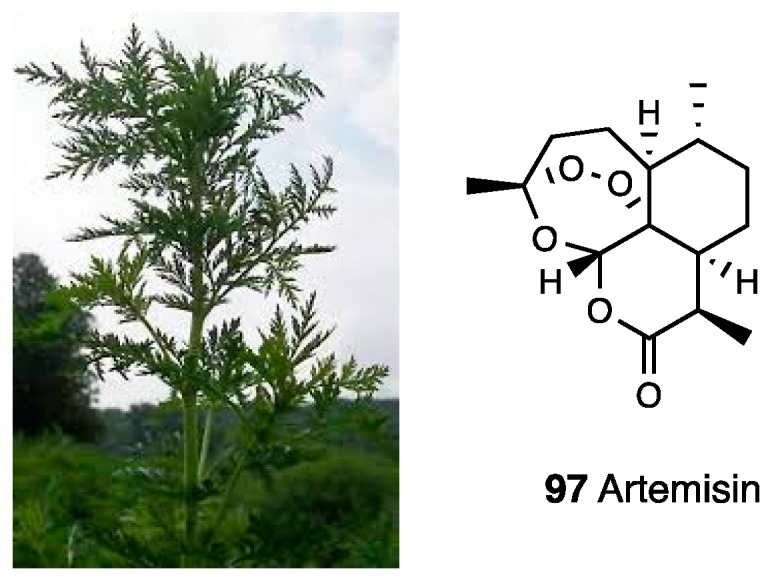
*Artemisia annua* and Artemisinin structure (**97**).

*Artemisia annua* L. is an Asiatic medicinal plant that was widely spread after being imported in Europe and the U.S. In ancient times, it was mainly used to treat fever and chills caused by malaria infection but was also used for tuberculosis, scabies, and dysentery. In 1971, artemisinin, the most active pharmacological compound of *Artemisia annua* against malaria, was isolated. Chemically, it is an endoperoxide sesquiterpene lactone (**97**, Figure 43).

Over the years, it was studied in other diseases for its immunoregulatory, anti-inflammatory, analgesic, anti-cancer, and anti-bacterial activities. De Faveri Favero et al. firstly tested the sesquiterpene lactone-enriched fraction from *Artemisia annua* leaves (1.72% of artemisin and 0.31% of deoxiartemisinin) in doses ranging from 30 to 100 mg/kg, by i.p. administration and in a formalin test, where 100 mg/kg exhibited a significant antinociceptive effect in the second phase in particular. These results were also corroborated by those obtained via CFA-induced inflammation, both in acute (4 h after CFA injection) and sub-chronic phases (24 h after CFA injection). They also demonstrated the antiedematogenic action of the fraction in carrageenan-induced paw edema, and the antinociceptive effect in the tail flick test, demonstrating opioid involvement due to naloxone’s pretreatment inhibition of an antinociceptive effect [228]. Ying M. et al. focused their experiment on a P2X_4_ purinergic receptors, which is expressed in satellite glial cells and dorsal root ganglion neurons—both involved in neuropathic pain conditions [229]. They demonstrated that artemisinin, at dose of 5 mg/kg i.p., exhibited a significantly antiallodynic effect starting from 7 days after CCI and summed up to an increase of thermal withdrawal latency, a reduction of both P2X_4_ mRNA level receptors, and protein expression in the dorsal root ganglion of CCI rats. They also found a reduction of P2X_4_ receptors and GFAP co-expression in the artemisinin-treated group compared to the CCI vehicle group [230]. On the other hand, Dehkordi et al. found GABA_A_ receptor involvement in the artemisinin (10 mg/kg i.p.) mechanism of action, because only bicuculline’s pretreatment significantly reverted its antinociceptive effects in an acetic acid-induced writhing test [231].

### 3.27. Sitosterole and Wrightiadione of Wrightia coccinea

**Figure 44:**
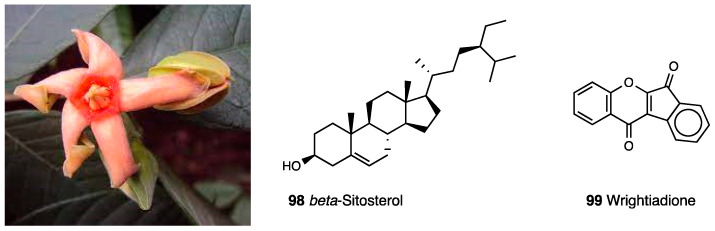
*Wrightia coccinea* and active constituents’ structure (**98** and **99**).

*Wrightia coccinea* (Roxb. Ex Hornem, Figure 44) is an indigenous plant (the less investigated in the *Wrightia* genus) of Southern Asia, also found in tropical Africa and China, and is widely used in folk medicine to treat snakebites, psoriasis, chest infections, and colic.

Its bark and seed pulp extract were studied by Jannat et al. to characterize their principal phytochemical compounds and their pharmacological proprieties [230]. They compared 200 mg/kg and 400 mg/kg methanolic extracts of bark and fruit coats, orally administered both in mice tail immersion tests to evaluate central analgesic activity and in acetic acid-induced writhing tests to evaluate peripheral analgesic activity. In both tests, the extracts exhibited a dose-dependent analgesic action. Considering phytochemical compounds, they found that β-sitosterol (**98**) and wrightiadione (**99**, Figure 44) showed better affinity vs. MOR and COX-2 enzymes, suggesting that they mainly determined the analgesic effect of the extracts [232], in line with other studies [233] on phytochemical compounds isolated by other *Wrightia* species.

### 3.28. Thymoquinone 

**Figure 45:**
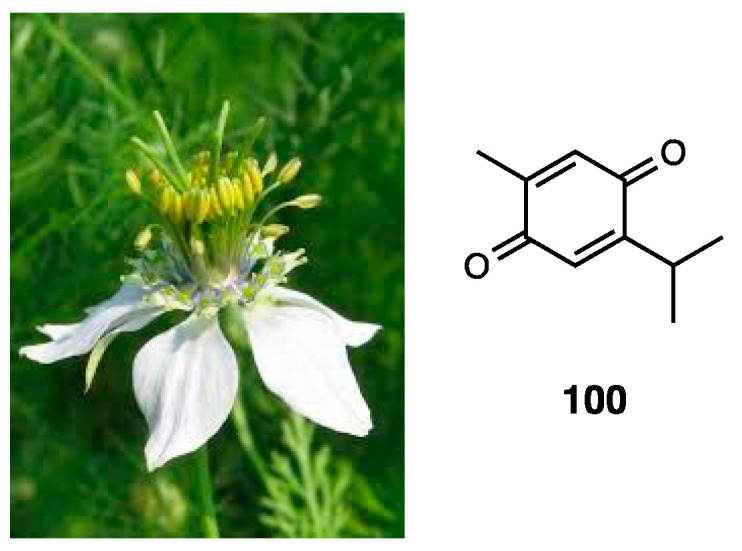
*Nigella sativa* and thymoquinone structure (**100**).

*Nigella sativa*, also known as black cumin due to the color of seeds, is one of the most popular medicinal plants (Figure 45). Belonging to the Ranunculaceae family, it is an annual plant that grows in different regions of Europe and Asia. In ancient medicine, its seed oil was used to treat abscesses, nasal ulcers, and asthmatic diseases like bronchospasm. *Nigella sativa* was also used as an analgesic, diuretic, and hepatoprotective agent and for energy recovery.

Considering seed phytochemical compounds, several studies indicate alkaloids (nigellimine-*N*-oxide, nigellicine and nigellidine), thymol, limonene, carvacol, *p*-cymene, and thymoquinone (**100**, Figure 45), which also is the most active compound. Literature studies indicate its hepatoprotective, anti-inflammatory, and nephroprotective actions [234,235,236,237].

Different studies indicate the inhibition of cyclooxygenase, 5-lypooxygenase, and leukotriene B4 but also of iNOS and MMP-1, MMP-3, and the reduction of pro-inflammatory cytokines such as IL-6 and TNF-α [238,239,240], confirming its anti-inflammatory activity. Through oral administration, Abdel-Fattah et al. firstly evaluated the antinociceptive effect of *Nigella sativa* oil (50–400 mg/kg) and tymoquinone (2.5–10 mg/kg, also i.p. injected 1–6 mg/kg). They discovered a significant antinociceptive effect of *Nigella sativa* oil in thermal, mechanical, and chemical nociceptive tests and the supraspinal action of tymoquinone mediated by the opioid system—probably by MOR and KOR [241]. In CCI test, Celik et al. evaluated the tymoquinone analgesic effects (100, 200 and 400 mg/kg p.o. and i.p.) demonstrating an anti-allodynic, not dose-dependent effect, which was not reverted by an opioid antagonist pre-treatment [242]. Their results, added with others obtained by Amin B., were in accordance with Abdel-Fattah et al., who discovered a supraspinal opioid involvement in tymoquinone action—only in the first phase of formalin tests—anti-inflammatory, and antioxidant effects in late phases and in neuropathic pain conditions [243].

### 3.29. (–)-Linalool

**Figure 46:**
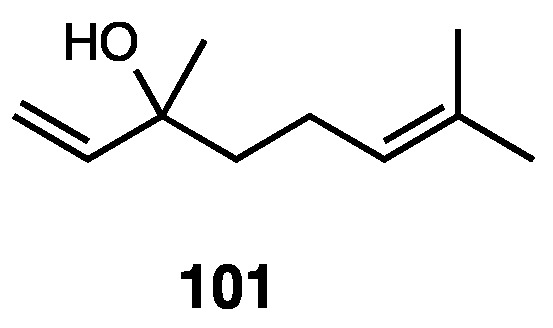
(–)-Linalool structure (**101**).

(–)-Linalool, a natural enantiomer monoterpene (**101**, Figure 46), is a widely volatile compound found in the essential oils of several plant such as *Citrus bergamia*, *Lavandula*, and *Jasminum,* and it is traditionally used to treat acute and chronic disease thanks to its anti-bacterial, anti-convulsant, and anxiolytic actions.

Peana et al. firstly described (–)-linalool anti-inflammatory action at a s.c.-injected dose of 50 mg/kg in carrageenan-induced edema as well as an anti-nociceptive effect both in acetic acid-writhing and hot plate tests, which was antagonized by naloxone and atropine pretreatment, showing opioid and cholinergic neurotransmission involvement [244,245]. Successively different mechanisms of action to explain linalool anti-nociceptive effects, like NMDA and adenosine receptor antagonism [246], were proposed. In neuropathic pain conditions, Berilocchi L. et al. firstly found that only repeated s.c. administration (100 mg/kg for 7 days) attenuated mechanical allodynia, starting from 3 days after L5 SNL, with a descreasing effect around 14 days after SNL, suggesting an anti-allodynic role for (-)-linalool only in the early phase of central sensitization. On the other hand, they did not find any significant changes in several pro-inflammatory cytokine’s levels and in glial activation [247]. Katsuyama et al. reported a better anti-nociceptive effect in both phases of a formalin test for (-)-linalool (2.5 and 5 μg) compared to *Citrus bergamia* essential oil (2.5, 5 and 10 μg). Moreover, they also confirmed opioid involvement in (-)-linalool antinociceptive action and discovered a peripheral site action trough naloxone methiodide administration [248]. Souto-Maior et al. focused their attention on linalool oxide, a minor component of essential oil formed from linalool natural oxidation, evaluating its antinociceptive effect at a dosage of 50, 100 and 150 mg/kg i.p. both in acetic acid-induced writhing tests and in both phases of the formalin test with linalool-comparable results [249]. After their results with the capsaicin test [250], Sakurada et al. evaluated the analgesic action of *Citrus bergamia* essential oil (5, 10 and 20 μg/paw) and linalool (2.5, 5 and 10 μg/paw) after i.pl. administration in SNL mice. The obtained results showed a significantly anti-allodynic effect starting from 5 min after injection for linalool, which were faster, stronger, and longer than oil, especially at 5 and 10 μg/paw. Moreover, they also discovered the inhibition of spinal ERK phosphorylation in bergamote essential oil and linalool anti-allodynic mechanism [251].

### 3.30. Zerumbone

**Figure 47:**
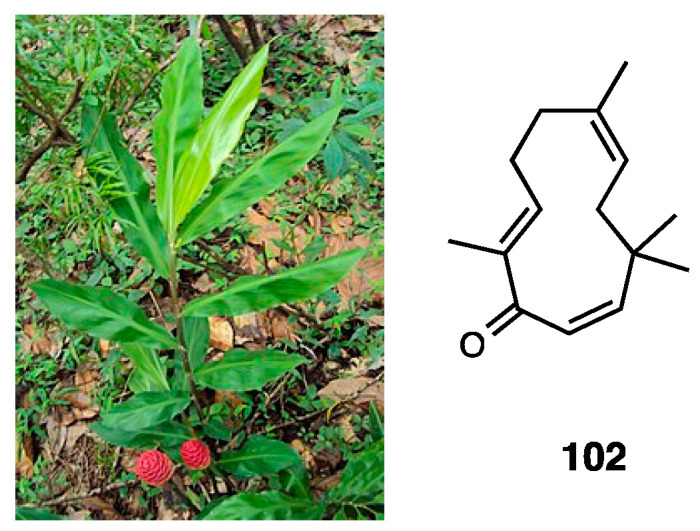
Structure of zerumbone (**102**), the major component from *Zingiber zerumbet*.

Zerumbone is the main component isolated from *Zingiber zerumbet,* a typical plant found in Malaysia rainforests. Chemically, it is a sesquiterpenoid (**102**, Figure 47), and it is used to treat stomachache, swelling, and muscle sprain.

Several studies reported its anti-inflammatory activity related to cyclooxygenase-2 inhibition. Firstly, Sulaiman et al. evaluated a dose-dependent antinociceptive effect both in acetic acid-induced abdominal writhing and in hot plate tests, suggesting a peripheral and central antinociceptive effect. They also found that naloxone pre-treatment significantly reverted its effect, indicating an opioid involvement [252]. Later, they confirmed its anti-inflammatory action on acute and chronic inflammation mice models [253]. In neuropathic pain conditions (CCI model), Zulazmi et al. firstly demonstrated the dose-dependent anti-allodynic and anti-hyperalgesic effect of zerumbone at doses of 10, 50 and 100 mg/kg i.p., which were probably related to the reduction of pro-inflammatory cytokines levels such as TNF-α and the desensitization of TRPV1 and TRPA1 channels [254,255,256]. Zerumbone anti-inflammatory effects in a CCI model were also confirmed by Gopalsamy et al., who also found a reduction of IL-6 and IL-1ß levels in the treated group [257]. They also confirmed both opioid system and K^+^ channel involvement in zerumbone’s analgesia in CCI-induced neuropathic pain [258,259]. On the other hand, Chia et al. focused their attention on cannabinoid and PPARs as the pharmacological targets of zerumbone’s analgesic action in CCI neuropathic pain, confirming molecular docking studies [260]. Previously, they also demonstrated that the anti-allodynic and anti-hyperalgesic effects of zerumbone treatment was related to a descending serotonin system, evaluated via serotonin depletion and with different subtype receptor antagonists [261].

### 3.31. Salvia officinalis

**Figure 48:**
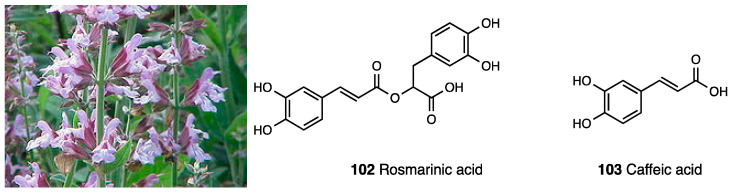
*Salvia officinalis* and structure of Rosmarinic acid and Caffeic acid (**102** and **103**).

*Salvia officinalis* (Figure 48) has been widely used for the antioxidant and anti-inflammatory properties of some of its active ingredients. The main components of *Salvia officinalis* leaves and hydroalcoholic extract are flavonoids and phenolic acids, in particular Rosmarinic acid (Ros) and Caffeic acid (Caf), respectively (**103**, **104**, Figure 48). They have a variety of pharmacological activities, including a proven analgesic effect; therefore, they have been recently studied in neuropathic pain models. The antinociceptive effects of the hydroalcoholic extract of *Salvia officinalis* were demonstrated in vincristine-induced neuropathic pain in mice in comparison with morphine, suggesting that *Salvia officinalis* extract could be useful in the treatment of peripheral neuropathic pain [262].

In a study conducted by Gabbas et al. [262], *Salvia officinalis* extract (100 and 200 mg/kg, p.o.), Ros (10 and 20 mg/kg, i.p.), Caf (30 and 40 mg/kg, i.p.), and Clomipramine (Clo, 5 mg/kg, i.p., a positive control) were given for 21 days after surgery in a CCI model, and these significantly and intermediately increased the reaction time to pain in animals 7 days after the induction of neuropathic pain and for the following 21 days [263]. It was also proven that *Salvia officinalis* extract exerts its analgesic activity through interaction with the opioid system. Bauer et al. [264] previously reported that *Salvia officinalis* extract compounds have analgesic effects via COX_2_ inhibitory effects. Caffeic acid has been proven to relieve neuropathic pain through the inhibition of proinflammatory cytokine expression such as IL-1β, IL-6, and TNF [265]. The analgesic efficacy of *Salvia officinalis* extract in neuropathic pain could be attributed to its anti-inflammatory activity. This hypothesis is corroborated by the observation that treatment with *Salvia officinalis* extract and its major compounds increased RBC levels and lowered CRP serum levels, indicating the remission of inflammation. Additionally, *Salvia officinalis* extract, Ros, and Caf promoted the motor function recovery of injured sciatic nerves and axonal regeneration, which was observed through an increase in the sciatic function index and histopathological analyses. These beneficial effects were attributed to its antioxidative, anti-inflammatory, and neuroprotective properties.

## 4. Discussion and Conclusions

Natural products, often characterized by enormous scaffold diversity and structural complexity, offer advantages and challenges for the drug discovery process. Natural product drug discovery is an articulated process. In the beginning, it consists of extraction from natural sources. This is a crucial phase, since the method of choice determines which compound classes will be present in the extract; for instance, polar solvents imply a higher percentage of polar compounds in the crude extract. Performing the extraction with several solvents at different polarities could maximize the diversity of the extracted natural products. Then, the biological screening of ‘crude’ extracts could identify a bioactive ‘hit’ extract, which is further fractionated to isolate bioactive compounds. After bioactive compound identification, molecular target identification follows. At this stage, a hit or lead compound could emerge that could be semi-synthetically optimized for its pharmacodynamic/pharmacokinetic properties.

In this review, we summarize recent efforts in natural product-based drug discovery in the therapeutic field of pain management, taking on the challenge of showing several compounds, which are different in structure and structural complexity, that could represent hit or lead compounds for further medicinal chemistry optimization.

Compared to classical synthetic drugs used for pain management, most of the bioactive compounds listed in this review have a higher molecular mass, higher numbers of H-bond acceptors and donors, higher hydrophilicity, fewer halogen atoms, and—for some of them—a lack of basic nitrogen and greater molecular rigidity.

For instance, Herkinorin is a potent MOR/KOR agonist derived by an intensive SAR study from Salvinorin A, the major constituent of *Salvia divinorum* Epling, and Jativa-M—both featured for their lack of positive charge nitrogen, which is a crucial structural requirement for opioid receptor interaction. Collybolide is also a non-nitrogenous KOR agonist with a higher effect/side effect ratio. Other non-nitrogenous bioactive compounds effective for persistent inflammatory pain are the primary active components of *Corydalis yanhusuo*, L-tetrahydropalmatine (l-THP), and protopine.

Moreover, different active principles examined showed a G-protein-biased profile, which is an improved approach to search for more tolerated drug candidates. Kurkinorin indeed was a biased MOR agonist as was Mitragynine, 7-OH-mitragynine and Corydaline. Nevertheless, Collybolide was instead a biased KOR agonist.

In conclusion, natural products represent an important tool for the discovery of scaffolds that can be developed or used as starting points for optimization into drugs. The “nature” laboratory continually contributes to knowledge underlying nociceptive modulation and remains a promising pool for the discovery of scaffolds with high structural diversity as well as various bioactivities that can be directly developed or used as starting points for optimization into novel drugs.

However, further research is needed to better establish the efficacy of different active principles with analgesic activity in humans so that many of these natural molecules can be useful for the development of new analgesics to continue making major contributions to human health and longevity.

## Figures and Tables

**Table 1:** Natural plant source, active principles, and synthetic analogues described in the review.

Natural Source		Active Principles	Synthetic Analogues
*Salvia divinorum*	(Lamiaceae)	Salvinorin A	Herkinorin Kurkinorin
*Mitragyna speciosa*	(Rubiaceae)	Mitragynine, 7-OH-Mitragynine, Mitragynine pseudoindoxyl	MGM-16 MGM-9
*Collybia maculate*	(Tricholomataceae)	Collybolide, 9-Epicollybolide	
*Corydalis yanhusuo* *Corydalis bungeana*	(Papaveraceae)	Corydine, Corydaline, Corynoline, L-tetrahydropalmatine (l-THP), Protopine, Dehydrocorydaline	Dehydrocorybulbine (DHCB)
*Himenaea cangaceira*	(Fabaceae)	Germacrene D, α-Humulene	
*Ageratum conyzoides*	(Asteraceae)	5,6,7,3′,4′,5′-hexamethoxyflavone, Nobiletin, 5′methoxynobiletin, Eupalestin	
*Arrabidaea brachypoda*	(Bignoniaceae)	Brachydin A, Brachydin B, Brachydin C	
*Nelumbo nucifera*	(Nymphaeaceae)	*N*-methylcoclaurine, Coclaurine, *O*-Methylcoclaurine Neferine,	
*Clinacanthus nutans*	(Acanthaceae)	Gallic acid, Caffeic acid, Ferulic acid, Vitexin, Apigenin	
*Azadirachta indica*	(Meliaceae)	Azadirachtin A	
*Vitex megapotamica*	(Lamiaceae)	*p*-Coumaric acid, Isoquercitrin, Naringenin, Caffeic acid	
*Salvia wagneriana*	(Lamiaceae)	(–)-Hardwickiic	
*Algrizea minor*	(Myrtaceae)	βPinene, αPinene, Germacrene D, Bicyclogermacrene, (E)-Caryophyllene, Limonene	
*Buddlejia globosa*	(Buddlejiaceae)	Verbascoside	
*Stachytarpheta* *cayennensis*	(Verbenaceae)	Ipolamiide, verbascoside	
*Tabernaemonta* *divaricata*	(Apocynaceae)	Conolidine	DS39201083, DS54360155, DS34942424
*Spinacia oleracea*	(Amarantacee)	Rubiscolin-6, Rubiscolin-5	
*Maytenus imbricata*	(Celastraceae)	Tingenone	
*Choisya ternata*	(Rutaceae)	Ternanthranin	
*Mansoa alliacea*	(Bignoniaceae)	Apigenin	
*Amomum subulatum*, *Boesenbergia pandurata*, *Alpinia rafflesiana*, *Alpinia katsumadai*, *Alpinia henryi*, *Campomanesia adamantium*	(Zingiberaceae) (Myrtaceae)	Cardamonin	
*Coptis* and *Berberis* species	(Berberidaceae)	Berberine	
*Aconitum* species	(Ranunculaceae)	Lappaconitine	
*Lantana camara*, *Lisgustrum lucidum*, *Rosmarinus officinalis*	(Verbenaceae) (Oleacee) (Lamiaceae)	Oleanolic acid	
*Myracrodruon* *urundeuva*	(Anacardiaceae)	Urundeuvine A, B and C	
*Artemisia annua*	(Asteracee)	Artemisin	
*Wrightia coccinea*	(Apocynaceae)	β-Sitosterol Wrightiadione	
*Nigella sativa*	(Ranunculaceae)	Thymoquinone	
*Citrus bergamia*, *Lavandula*, *Jasminum*	(Rutacee) (Labiate) (Oleaceae)	(–)-Linalool	
*Zingiber zerumbet*	(Zingiberaceae)	Zerumbone	
*Salvia officinalis*	(Labiate)	Rosmarinic acid, Caffeic acid	

**Table 2:** Binding affinities and functional activities for Salvinorin A and its analogues.

	*Ki* (nM) ± S.D.			EC_50_ ± S.D. (*E_max_* ± S.D.) ^a^			
Compound n	MOR	DOR	KOR	MOR	DOR	KOR	REF
**1** Salvinorin A	>10,000	>10,000	18.74 ± 3.38	>10,000	>10,000	7 (104 ± 7)	[26]
**2** Salvinorin B	>10,000	>10,000	>10,000	>10,000	>10,000	>10,000	[26]
**13** Herkinorin	12 ± 1	1170 ± 60	90 ± 2	500 ± 140 (130 ± 4)	>10,000	1320 ± 150 (140 ± 2) ^b^	[47]
**14** Herkamide	3.1 ± 0.4	810 ± 30	7430 ± 880	360 ± 60 (134 ± 5)	N.D.	N.D. ^b^	[47]
**15** Kurkinorin	N.D.	N.D.	N.D.	1.2 ± 0.6	74 ± 10	>10,000	[48]

N.D. not determined; S.D. standard deviation. ^a^ Biological activity compared with DAMGO, SNC-80, and U50488. ^b^ Biological activity compared with U69593.

**Table 3:** Binding affinities and functional activities for mitragynine and its analogues.

	*pKi* ± S.D. ^a^			
Compound n	MOR	DOR	KOR	REF
**16** Mitragynine	8.14 ± 0.28	7.22 ± 0.21	5.96 ± 0.22	[58]
**17** 7-OH-Mitragynine	7.87 ± 0.16	6.81 ± 0.19	6.91 ± 0.07	[58]
**18** Mitragynine pseudoindoxyl	10.06 ± 0.39	8.52 ± 0.22	7.10 ± 0.32	[58]
**20** 9-hydroxycorynantheidine	7.92 ± 0.05	4.51 ± 0.15	5.53 ± 0.07	[58]

S.D. standard deviation. ^a^ Radioligand [^3^H]DAMGO, [^3^H]DPDPE, and [^3^H]U69593.

**Table 4:** Binding affinities and functional activities for corydine and corydaline.

	*Ki* (μM) ± S.D. ^a^			EC_50_ ± S.D. *(E_max_* ± S.D.) ^b^	
Compound n	MOR	DOR	KOR	MOR	REF
**26** Corydine	2.82 ± 0.61	N.D.	N.D.	0.51 ± 0.11 (102 ± 6)	[80]
**27** Corydaline	1.23 ± 0.29	N.D.	N.D.	1.50 ± 0.44 (104 ± 6)	[80]

N.D. not determined; S.D. standard deviation. ^a^ Radioligand [^3^H]DAMGO. ^b^ Biological activity compared with DAMGO.

**Table 5:** Binding affinities of *Nelumbo nucifera* active principles.

	*Ki* (μM) ± S.D. ^a^			
Compound n	MOR	DOR	KOR	REF
**47** *O*-methylcoclaurine	2.0 ± 0.3	22.6 ± 3.9	3.5 ± 0.3	[123]
**48** *N*-methylcoclaurine	2.82 ± 0.61	20.1 ± 3.1	0.9 ± 0.1	[123]
**49** Coclaurine	5.0 ± 0.7	21.1 ± 2.0	2.2 ± 0.2	[123]
**50** Neferine	1.8±0.2	0.7 ± 0.1	3.3 ± 0.4	[123]

S.D. standard deviation. ^a^ Radioligand [^3^H]DAMGO, [^3^H]DPDPE, and [^3^H]U69593.

## Data Availability

The data presented in this study are available on request from the corresponding author.

## Abbreviations

The following DMF Dimethyl fumarate; FDA food and drug administration; EMA European medicinal agency; MOR mu-opioid receptor; DOR delta-opioid receptor; KOR kappa-opioid receptor; i.t. intrathecal; i.p. intraperitoneal; i.c. intracisternal; p.o. oral; GPI guinea pig ileum; s.c. subcutaneous; i.c.v. intracerebroventricular; PGE2 prostaglandin E2; LPS lipopolysaccharide; MVD mouse vas deferens; SNL sciatic nerve ligation; HPLC high performance liquid chromatography; DHCB dehydrocorybulbine; l-THP L-tetrahydropalmatine; Sig-1R sigma1 receptor; CCI chronic constriction injury; GC-MS gas chromatography-mass spectroscopy; UHPLC/MS ultra-high performance liquid chromatography-mass spectroscopy; i.g. intragastric; i.pl. intraplantar; HPLC–PDA high-performance liquid chromatography-photodiode array detection; TRPM8 transient receptor potential melastatin 8; ASIC acid-sensing ion channel; TRPV1 transient receptor potential vanilloid 1; TRPA1 transient receptor potential ankyrin 1; IL-6 interleukin-6; CNS central nervous system; BTIQ benzyltetrahydroisoquinoline; CB-1 cannabinoid-1; CB-2 cannabinoid-2; NMDA N-methyl-D-aspartate; PKC protein kinase C; PSNL partial sciatic nerve ligation; NA noradrenaline; DA dopamine; ROS reactive oxygen species; ACK3 chemokine receptor; 3D-QSAR Three dimensional-quantitative structure-activity relationships; CoMFA comparative molecular field analysis; CoMSIA comparative molecular similarity indices analysis; NO nitric oxide; COX-1 cyclooxygenase-1; COX-2 cyclooxygenase-2; PBQ 1,4-benzoquinone; 5-HT1A 5-hydroxytryptamine; STZ streptozotocin; mRNA messenger ribonucleic acid; TNF-α tumour necrosis factor-α; IL-6 interleukin-6; SOD superoxide dismutase; GPx glutathione peroxidase; CAT catalase; NF-κB nuclear factor κB; SAR structure–activity relationship; TDD transdermal drug delivery; GFAP glial fibrillary acidic protein; GABA_A_ gamma amino butyric acid-A; iNOS inhibitor nitric oxide synthetase; MMP-1 matrix metalloproteinase-1; MMP-3 matrix metalloproteinase-3; PPARs peroxisome proliferator-activated receptors; RBC Red Blood cells; CRP C reactive protein; CFA complete Freund’s adjuvant; Iba 1 ionized binding protein 1, SAP s.c.air pouch.
